# Global motion coherent deficits in individuals with autism spectrum disorder and their family members are associated with retinal function

**DOI:** 10.1038/s41598-025-11789-y

**Published:** 2025-08-02

**Authors:** Irene O. Lee, Dennis M. Fritsch, Maximilian Kerz, Jane C. Sowden, Paul A. Constable, David H. Skuse, Dorothy A. Thompson

**Affiliations:** 1 Behavioural and Brain Sciences Unit, Population Policy and Practice Programme, Great Ormond Street Institute of Child Health, University College London, London, UK; 2 Oceano Azul Foundation and Katapult Ocean, Baden Württemberg, Germany; 3 Cherry Health, Calgary, Canada; 4 Great Ormond Street Institute of Child Health, University College London and Great Ormond Street Hospital NIHR Biomedical Research Centre, London, UK; 5 College of Nursing and Health Sciences, Caring Futures Institute, Flinders University, Adelaide, Australia; 6 Great Ormond Street Institute of Child Health, University College London, London, UK; 7 Clinical and Academic Department of Ophthalmology, Great Ormond Street Hospital for Children NHS Trust, London, UK

**Keywords:** Global motion perception, Electroretinogram, Coherent motion, Retinal function, Magnocellular pathway, Autism family, Physiology, Psychology, Medical research

## Abstract

This study aims to evaluate if the reduced sensitivity to global motion observed in some individuals with autism spectrum disorder (ASD) is associated with altered retinal processing. Motion coherence thresholds were measured from individuals with ASD and their family members and compared to the test reference limits derived from control participants. The light adapted electroretinogram (ERG) a- and b-wave amplitudes and peak-times, and photopic negative response (PhNR) parameters were measured from the ASD individuals and their families and compared to those of controls. Abnormally high motion coherence thresholds were found in ASD probands and their family members compared to that in controls, particularly mothers. Altered retinal functions were found in ASD probands and their parents. The PhNR, a- and b-wave time-to-peak were significantly correlated with motion coherence thresholds. The altered retinal function was associated with the age, intelligence and autism severity of the ASD family members. There were associations between the motion coherence and ERG parameters, including smaller amplitudes of the PhNR, and longer time-to-peak of the a- and b-waves and time to the PhNR, compared to those with abnormal motion coherence thresholds. The results showed that global motion coherence deficits were associated with altered retinal function in ASD and their family members. The findings suggest that motion perception deficits follow a familial pattern and that affected mothers may have an increased risk of a child with ASD.

**Supplementary Information:**

The online version contains supplementary material available at 10.1038/s41598-025-11789-y.

## Introduction

Autism spectrum disorder (ASD) is a neurodevelopmental condition, characterised by deficits in the domains of social reciprocity, social communication, repetitive patterns of behaviour and atypical responses to sensory input or unusual interests in sensory aspects of the environment^1,2^. ASD affects approximately 1% of the population^1,3,4^ and is more commonly diagnosed in males, with a male-to-female ratio of 3:1^3,5^. ASD is highly heritable with estimates ranging between 64 and 91%^3,6^ and therefore the potential for the parents and their affected children to have differences in their motion coherence thresholds was explored in this study.

ASD is a lifelong disorder and the profound impact it has on communication and social interactions can be exacerbated by hyper- or hyposensitivity to sensory (auditory, visual, tactile) stimuli that negatively compound these social and communication domains^2,4,7,8^. Multiple visual sensory symptoms are reported by individuals with ASD^9–11^ including a relative insensitivity to detecting global motion^4^. A sense of global coherence for motion enables observers to perceive the overall direction of moving objects and is pivotal for interpreting dynamic sensory input^12,13^.

Global motion processing, involves integrating information from motion cues across space and time^14,15^, and depends on higher areas along the dorsal stream, primarily the middle temporal and medial superior temporal (MT/MST) complex, and extrastriate areas located in the intraparietal sulcus^4,16^. Another aspect of visual processing is local motion processing, which is lower order and involves the primary visual cortex (V1) that processes local elements of the visual scene before integration by the higher cortical areas^13^. Collectively these cortical areas contribute to overall global motion perception, integrating local motion signals into global precepts with the guidance of eye movements^17^. Atypical global motion processing has a negative impact on how an individual perceives and interacts with the world^4,18^. In ASD, poor global motion perception is widely though variably reported^19,20^ and may contribute to poor social interactions observed in these individuals.

Motion coherence detection is one measure of global motion perception^21^. In ASD, elevated motion coherence thresholds have been reported in various studies^22–25^, with few exceptions^4,26,27^. Psychophysical studies of motion coherence have varied extensively in their design complexity to understand the mechanisms of motion perception in adults over many years, but few tests are available for children. Studies using electrophysiological techniques which are easier to apply in children and young adults are less common, such as studying the electrical potentials of the eye using the electroretinogram (ERG) or visual evoked potentials (VEPs) in response to the onset of a moving target^28^. The ERG may reveal differences in signalling pathways in the retina that are common to the brain and could investigate whether early sensory function of the retina may also impact on motion processing^29,30^. The ERG waveform displays the changes in voltage over time produced by the retina in response to a brief flash of light under dark- or light-adapted (DA or LA) conditions^31,32^. The problem of visual motion detection has traditionally been cast in terms of the properties of retinal image features^33^. Smaller than average ERG amplitudes under DA and LA conditions have been reported in ASD^34–38^ suggesting differences in early retinal processing in ASD may impact on higher cortical processing involving the visual pathways. Another report observed altered ERG amplitudes in the parents and young siblings of probands diagnosed with ASD^39^.

In this study, we aimed to identify ASD individuals and their family members with elevated motion coherence thresholds compared to a control cohort and to examine whether motion coherence thresholds were related to functional measures of the retina using the LA-ERG.

## Results

The demographic information of the recruited participants for motion coherence test and electroretinogram is shown in Table 1.

**Table 1:** Participant demographic information for motion coherence threshold and the electroretinogram.

Mean ± SD		CTL Total	CTL ≤16	CTL 17–27	CTL > 28	ASD Total	ASD ≤16	ASD 17–27	ASD’s Sibling Total	ASD’s Sibling ≤16	ASD’s Sibling 17–27	ASD’s parent Total	ASD’s Father	ASD’s Mother
N	MC	194	85	44	65	40	31	9	17	10	7	18	6	12
	ERG	29	16	13	–	43	33	10	20	12	8			
% Male	MC	47%	49%	44%	48%	73%	74%	67%	24%	20%	29%	33%	100%	–
	ERG	48%	50%	46%	–	74%	79%	67%	30%	33%	25%	33%	100%	–
Age (year)	MC	24.2 ± 16.1	10.0 ± 3.1	22.7 ± 3.4	43.5 ± 11.0	15.0 ± 5.2^a^	12.7 ± 2.7^b^	23.0 ± 3.5	17.0 ± 6.3	13.6 ± 4.6^c^	22.0 ± 5.3	48.9 ± 4.9	48.7 ± 3.5	49.6 ± 5.7
	ERG	15.3 ± 4.6	11.7 ± 2.3	19.8 ± 1.9	–	14.8 ± 4.6	12.7 ± 2.7	23.0 ± 3.5	15.8 ± 4.6	13.1 ± 2.8	16.4±5.1	48.9 ± 4.9	48.7 ± 3.5	49.6 ± 5.7
Age median (year)	MC	20.0	10.0	23.0	45.0	14.2	13.2	23.6	16.3	13.5	23.0	50.2	49.9	50.2
	ERG	14.2	10.0	19.5	–	14.2	13.2	23.6	16.3	12.4	21.2	50.2	49.3	50.2
Age range (year)	MC	4–70	4–16	17–27	28–70	6–27	6–16	17–27	10–27	10–16	18–27	39–58	44–53	39–58
	ERG	6–24	6–16	17–24	–	6–27	6–16	17–27	8–27	8–16	18–27	39–58	44–53	39–58
FSIQ	MC	–	–	–	–	98.0 ± 19.4	95.6 ± 16.1	105.3 ± 27.0	–	–	–	–	–	–
	ERG	–	–	–	–	98.5 ± 19.4	95.8 ± 15.9	106.3 ± 25.6	–					
ADOS total	MC	–	–	–	––	11.9 ± 4.8	11.7 ± 4.8	12.6 ± 4.7	–	–	–	–	–	–
	ERG	–	–	–	–	11.8 ± 4.7	11.7 ± 4.8	12.1 ± 4.7	–					
Autism severity	MC	–	–	–	–	6.9 ± 1.8	6.9 ± 1.7	7.0 ± 1.9	–	–	–	–	–	–
	ERG	–	–	–	–	6.8 ± 1.8	6.9 ± 1.7	6.7 ± 2.1	–					
NMed	MC	–	–	–	–	6 (15%)	4 (13%)	2 (22%)	1 (6%)	0	1 (14%)	8 (44%)	1 (17%)	7 (58%)
	ERG	–	–	–	–	6 (14%)	4 (12%)	2 (20%)	1 (5%)	0	1 (13%)			
Iris colour index*	ERG	1.3 ± 0.1	1.3 ± 0.1	1.3 ± 0.1	–	1.3 ± 0.1^d^	1.2 ± 0.1^d^	1.2 ± 0.1^d^	1.3 ± 0.1	1.3 ± 0.1	1.3 ± 0.1	1.3 ± 0.1	1.3 ± 0.1	1.3 ± 0.1

Data are presented as mean ± standard deviation (SD).

*CTL* = Control, with no ASD proband in their first-degree family, *N =* Number of participants taking part in the motion coherence test and ERG test, *FSIQ* = Full Scale IQ, *ADOS total* = Autism Diagnostic Observation Schedule total score, *Autism Severity* = Autism severity score, *NMed* = Number of participants taking medications before testing.

*Iris colour index were measured from the RETeval device (LKC Technologies Inc, Gaithersburg, MD, USA) during the ERG testing and exported from the RFF extractor version 2.9.4.1.

^a^A significant difference between the mean age of Control total and ASD total (*p* < 0.001).

^b^A significant difference between the mean age of Control ≤16 and ASD ≤16 groups (*p* < 0.001).

^c^A significant difference between the mean age of Control ≤16 and ASD’s Sibling ≤16 groups (*p* < 0.001).

^d^The mean iris colour index of each ASD group was significantly different from that of the Control group (*p* < 0.05, for each Total, ≤16, 17–27 age group), and it is also applied when compared to the ASD’s parent groups (*p* < 0.05).

### Motion coherence test

**Fig. 1:**
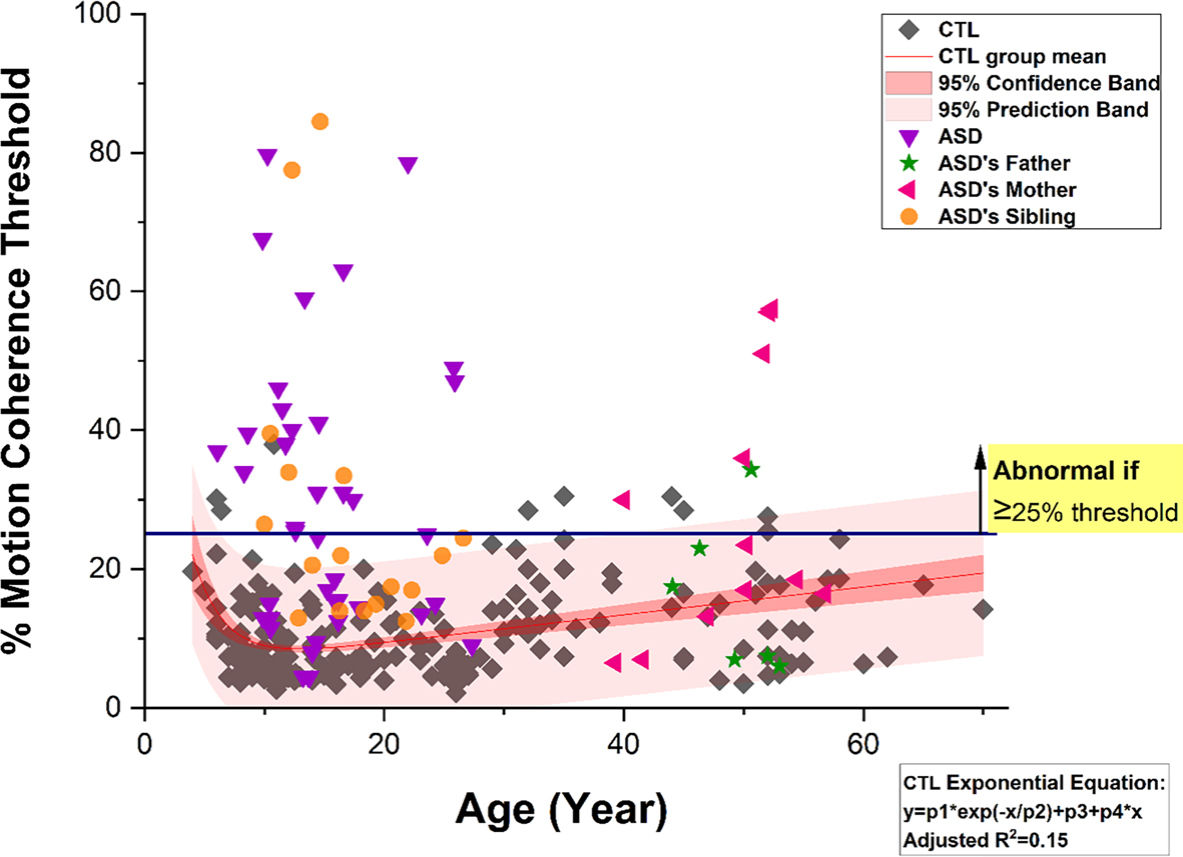
Percentage thresholds of motion coherence against age of the control individuals and autism family members. *CTL* = control individuals, *ASD* = autism spectrum disorder. Total Signal Dots were 1081. The red straight line presents the group mean, 95% confidence interval in dark red band and 95% prediction interval in light red band.

The motion coherence test was performed with black dots on a white background (BoW) and with white dots on a black background (WoB). The average motion coherence thresholds of BoW and WoB were used for comparison amongst the groups, as no significant differences between the two tests were found (*p* > 0.54, Supplementary Table S1). Overall, there was no linear correlation between the participant’s age and the mean motion coherence thresholds (*N* = 269, *r* = 0.053, *p* = 0.376). However, in the control group, the motion thresholds were age-dependent, and the threshold values were plotted against their ages in Fig. 1. The younger participants below 6 years old required higher thresholds and the thresholds were reduced exponentially with increasing age, as the visual system matures, until the age of 30 when threshold began to rise again. This changed considerably with age, and a significant increase of thresholds was observed in the older participants. To illustrate changes in motion coherence thresholds age was grouped into those ≤16 years old, those aged 17–27 and those aged from 28 to 70 years old, shown in Table 2. The motion coherence thresholds in mean ± SD (median) of the control individuals were 9.9 ± 6.4 (8.0), 7.9 ± 3.8 (7.0) and 14.4 ± 6.9 (13.3) for those ≤16 years old, 17 to 27 years old and above 28 years old respectively, with a significant difference amongst these age groups (*p* < 0.001). However, they fall within normal thresholds (i.e. under 25%) and the overall threshold in the whole control group was 10.9 ± 6.6 (8.8), with 95% CI between 10.0 and 11.8. Figure 1 illustrates the negatively exponential shaped skewed distribution to form a 95% confidence in: Percentage thresholds of motion coherence against age of the control individuals and autism family memberterval of 10.5% and 12.7% within a range of 2.2–41.0% thresholds and mean absolute deviation of 5.8%.

**Table 2:** Mean of percentage motion coherence detection thresholds of both tests (test with black dots on a white background and test with white dots on a black background) in ASD family groups and control age groups.

	Age group (year)	*N*	% Motion coherence threshold			*p*-value	
			mean ± SD	Median	95% CI	VS CTL	VS ASD
CTL	≤ 16	85	9.9 ± 6.4	8.0	8.5–11.3	< 0.001^ǂ^	*< 0.001*
	17–27	44	7.9 ± 3.8	7.0	6.8-9.0		*< 0.001*
	28–70	65	14.4 ± 6.9	13.2	12.7–16.1		--
	Total	194	10.9 ± 6.6	8.8	10.0-11.8	--	*< 0.001*
ASD	≤ 16	31	^ψ^28.7 ± 19.8	^ψ^25.5	21.7–35.7	*< 0.001*	0.74
	17–27	9	^ψ^31.3 ± 22.9	^ψ^25.0	16.3–46.3	*< 0.001*	
	Total	40	^ψ^29.2 ± 20.2	^ψ^25.3	22.9–35.5	*< 0.001*	--
ASD’s sibling	≤ 16	10	^ψ^36.5 ± 25.0	^ψ^30.0	21.0–52.0	*< 0.001*	0.31
	17–27	7	17.5 ± 4.3	17.0	14.8–20.2	*< 0.001*	0.14
	Total	17	^ψ^28.7 ± 21.0	22.0	18.7–38.7	*< 0.001*	0.93
ASD’s dad		6	15.9 ± 11.3	12.5	6.9–24.9	0.629	0.12
ASD’s mum		12	^ψ^25.1 ± 16.8	18.5	16.0-34.6	*< 0.01*	0.83
	Total	18	23.8 ± 17.2	18.0	15.9–31.7	*0.007*	0.33

CTL = Control who has no ASD in the first-degree family; N = number of individuals; % Motion coherence threshold = average of percentage motion detection coherence thresholds from black dots on a white background test and white dots on a black background test.

^ψ^Threshold ≥ 25% is abnormal. CI = Confidence interval; VS = compared with. ^ǂ^ANOVA comparison within group, however, all are normal thresholds.

Significant values are in italics.

**Fig. 2:**
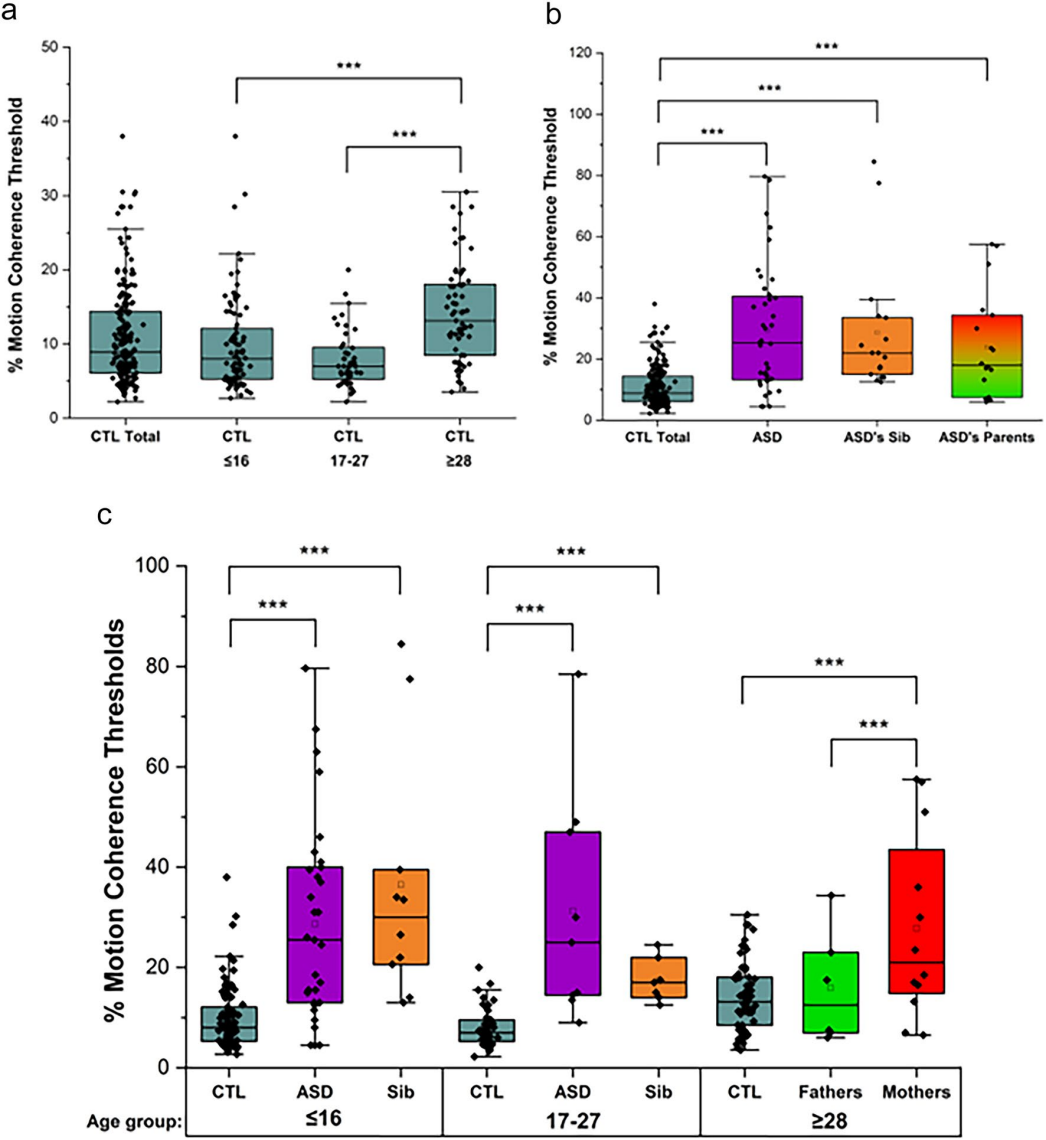
Box plots to show the elevated percentage thresholds of motion coherence of control group and the ASD family members. The percentage motion coherence thresholds on the y-axis were the percentage of the total signal dots and were average of positive and negative contrast WoB and BoW tests from white dots on a black background and black dots on a white background respectively. Figure 2a shows % coherence thresholds of Control in each age group; Fig. 2b shows % coherence thresholds of ASD family and control groups; Fig. 2c shows % coherence thresholds of each age group in different family member groups. CTL = Control; Fathers = ASD’s fathers; Mothers = ASD’s mother; Sib = ASD’s sibling. % Motion coherence thresholds ≥ 25% were considered abnormal. ***p < 0.01. Median line and mean (box shape) of each group are shown within each box plot.

Figure 2 shows the box plots of the percentage motion coherence thresholds for the control (Fig. 2a) and the ASD family group (Fig. 2b) by age (Fig. 2c). Compared to the control group, the ASD group, their siblings and mothers had significantly (*p* < 0.01) elevated motion coherence thresholds. The results have been compared between the same age groups with the other family groups, as well as between the age groups within the same group. The percentage thresholds [mean ± SD (median)] of the ASD probands for those ≤16 and 17–27 groups 28.7 ± 19.8 (25.5) and 31.3 ± 22.9 (25.0) respectively, which were statistically significant (both p-values < 0.001) compared to that of the control cohorts. A similar finding was found within the siblings of the ASD group where their motion coherence detection thresholds were higher 36.5 ± 25.0 (30.0) and 17.5 ± 4.3 (17.0) in the ≤16 and 17–27 age groups respectively (both p-values < 0.001). The motion coherence thresholds in ASD’s mothers were 25.1 ± 16.8 (18.5), which was significantly higher than the control group aged 28–70 (*p* < 0.01), whereas the motion coherence thresholds of the ASD’s fathers were 15.9 ± 11.3 compared to control group aged 28–70 were non-significant (*p* = 0.63). Overall, no statistically significant differences of motion coherence thresholds between sex in all the groups were found, except in the results of the ASD’s siblings aged between 17 and 27, for which the motion thresholds of female siblings were significantly lower than that of male siblings but both sexes had normal thresholds and the sample size of male siblings was only 2 (Supplementary Table S2).

Table 3 lists the percentage of individuals with abnormal motion coherence thresholds in each age group of the control and different ASD family groups. 9 control individuals (4 males and 5 females), that is 4.6%, out of 194 controls required more than 25% of total signal dots for motion coherence detection, of which 6 (9.2%) of the older controls (aged over 28) resulted in abnormal motion coherence. This is significantly different in the ASD probands, where over 55% of ASD participants, 15 males and 7 females, had higher motion coherence thresholds with no significant differences between the age groups. A similar result was observed in the siblings of the ASD group aged ≤16 years with a motion coherence threshold of 60% (1 male and 5 female participants) and with 41.2% of the total number of ASD’s sibling group having high motion coherence thresholds. 5 out of 12 mothers (41.7%) also required higher than 25% motion coherence thresholds and only 1 father out of 6 (16.7%) had an elevated motion coherence threshold (see Table 3).

**Table 3:** Percentage of the number of individuals with abnormal motion coherence thresholds in each age group of the control and different ASD family groups.

	Age group (year)	N	Abnormal motion coherence threshold	
			% of group	M/F count
CTL	≤16	85	3.5	2/1
	17–27	44	0	0/0
	28–70	65	9.2	2/4
	Total	194	4.6	4/5
ASD	≤16	31	54.8	11/6
	17–27	9	55.6	4/1
	Total	40	55.0	15/7
ASD’s sibling	≤16	10	60.0	1/5
	17–27	7	14.3	1/0
	Total	17	41.2	2/5
ASD’s parents	ASD’s Father	6	16.7	1
	ASD’s Mother	12	41.7	5
	Total	18	33.3	6

*CTL* = Control group, *N* = number of subjects in each group, *M* = Male, *F* = Female.

**Fig. 3:**
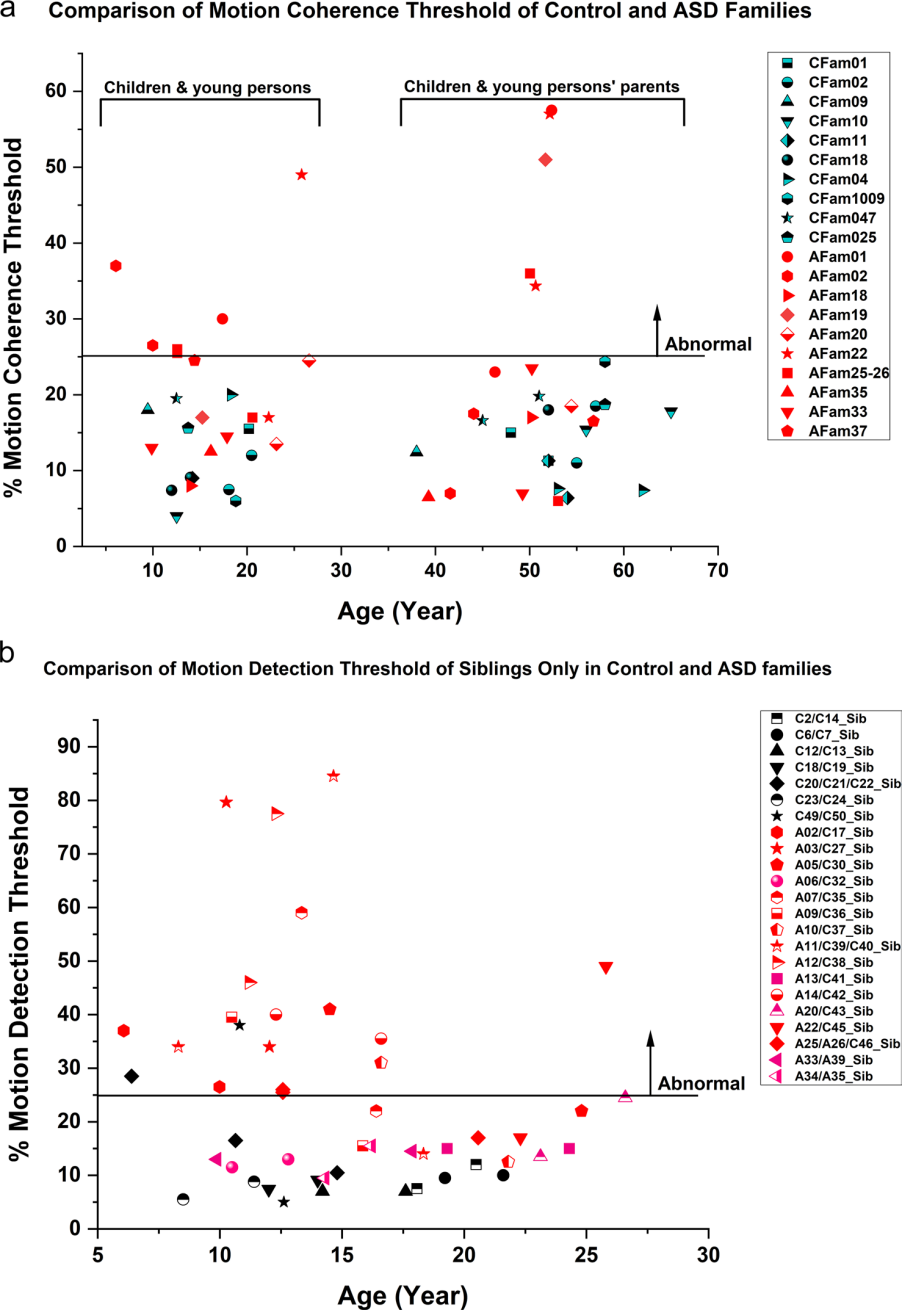
Comparison of the motion coherence thresholds between Control and ASD family members. Each family has the same shape and colour. (**a**) Comparison of the percentage motion detection thresholds of 10 Control Families (CFam) and 10 Autism Families (AFam), including their parents and siblings. (**b**) Comparison of the percentage motion detection thresholds of siblings only. Black signs represent control siblings from 7 families and red are from 16 ASD probands’ siblings. The codes start with C or A represents those with or without ASD respectively.

Figure 3a shows the scatter plot of the motion coherence thresholds for the 10 control and 10 ASD families. All the children and parents in the control groups had normal motion coherence thresholds (that were < 25%). However, amongst the 10 ASD families, 4 ASD families had elevated motion coherence thresholds, including a pair of siblings (ASD proband and ASD’s control siblings in Afam02), both parents and probands (Afam22), and another 2 families with 1 parent and ASD proband (Afam01 and Afam25-26). When differences of the motion coherence thresholds were compared between the siblings only in the control and ASD families (Fig. 3b), then out of the 7 groups of siblings in the control families, only 2 children in separate families were above the 25% thresholds (C21 and C49), and their siblings and the other 5 pairs of siblings had all normal thresholds. Amongst the 16 ASD families (at least 1 ASD per family), normal motion thresholds were observed in 4 pairs of siblings (A06/C32_Sib, A13/C41_Sib, A33/A39_Sib and A34/A35_Sib). 6 pairs of ASD probands and their siblings required significantly higher motion thresholds (A02/C17_Sib, A03/C27_Sib, A11/C39_Sib, A12/C38_Sib, A14/C42_Sib and A25/A26_Sib). In another 6 pairs of ASD and their non-ASD siblings, 4 ASD probands (A05, A07, A10 & A22) and 2 ASD’s siblings (C36, C43) in separate families had abnormally higher motion coherence thresholds.

#### Motion coherence deficits were not associated with IQ, autism severity, comorbidities and medications

The measures of IQ scores, autism severity and comorbidities were investigated for their effects on motion coherence deficits in the ASD probands. There were no correlations between the motion coherence thresholds with the full-scale IQ score (*N* = 33, *r*=− 0.256, *p* = 0.151, see Supplementary Table S3a), ADOS score (*N* = 34, *r*=− 0.016, *p* = 0.928), or autism severity score (*N* = 34, *r* = 0.020, *p* = 0.912) of the ASD probands. Furthermore, the motion coherence deficits were very unlikely related to the comorbidities of the ASD probands with no significant differences of motion coherence thresholds between participants with or without comorbidities (*N* = 40, χ^2^ = 0.494, *p* = 0.482, see Supplementary Table S3b).

Since there was no one taking any medications related to central nervous system (CNS) in the control group, we only evaluated the effects of CNS medications amongst the ASD family members, 21 of them (28%) had taken CNS medications before testing, that is including ASD’s siblings and parents. 12 (57%) who took CNS resulted abnormal motion coherence thresholds compared to those without taking it (46%). The likelihood of CNS medicine on motion detection deficit was not significant (*N* = 75, χ^2^ = 0.712, *p* = 0.399, see Supplementary Table S3b), suggesting that CNS medications are unlikely to have affected the motion perception.

### Electroretinogram in families

**Fig. 4:**
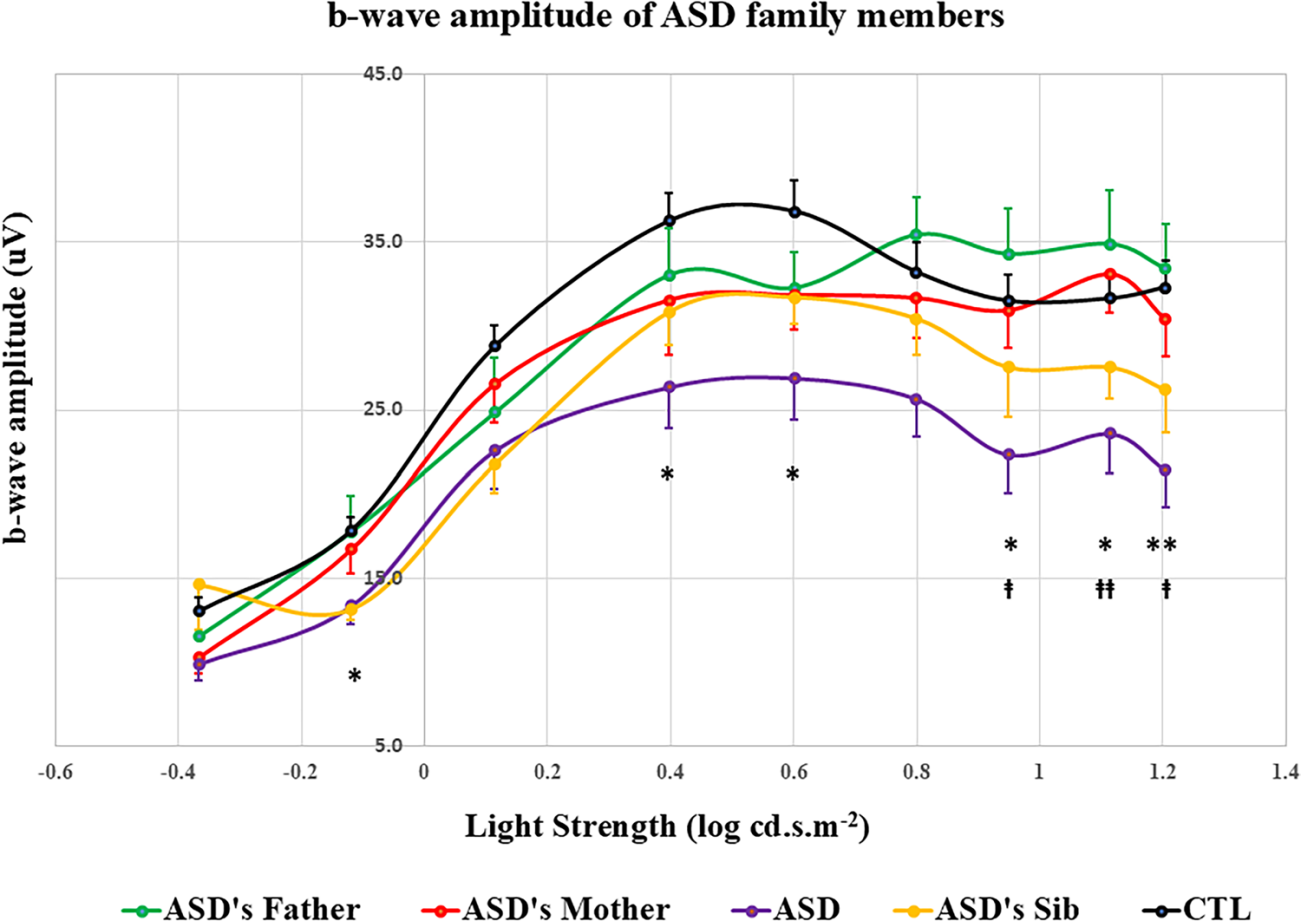
Comparisons of b-wave amplitudes ASD probands with their family members and with the control group. All data points are means and standard error bars. *ASD* = proband with autism spectrum disorder, *Sib* = ASD’s sibling, *CTL* = Control group. b-wave amplitudes of ASD probands were significantly lower than that of controls, *p < 0.05, **p < 0.01; significant differences of b-amplitudes between ASD and ASD’s father group, ^ǂ^*p* < 0.05; ASD proband’s b-wave amplitudes were significantly lower than ASD’s mother group, ^ǂǂ^*p* < 0.05.

The means of the a- and b-wave amplitudes of all ASD’s parents and siblings responding to all flash strengths were not significantly different from that of the control participants after Bonferroni corrections (see Fig. 4 and Supplementary Figure S1). However, the a-wave amplitudes of the ASD probands were significantly lower than that of control as well as those of ASD’s mothers at the flash strength of 1.204 log cd.s.m^− 2^ (Supplementary Figure S1). Furthermore, the b-wave amplitudes of ASD probands were significantly attenuated compared to the control measures at the flash strengths of -0.119, 0.4, 0.6, 0.95, 1.114 (all *p* < 0.05, see Fig. 4) and 1.204 log cd.s.m^− 2^ (*p* < 0.01). They were also significantly lower than the b-wave amplitudes of ASD’s fathers at 0.95 and 1.204 log cd.s.m^− 2^ (*p* < 0.05) and of ASD’s mothers at 1.114 log cd.s.m^− 2^ (*p* < 0.01). The photopic hill of the parents showed a similar trajectory to the control group for the peak and plateau phase^40^. Further studies with a larger population will be required to confirm these findings in the future.

For the time-to-peak of the a-waves, Bonferroni tests have shown no significant differences between ASD probands and the family members, as well as with the controls (all p-values > 0.06). For the b-wave time-to-peak, there were multiple statistically significant differences between the control and ASD’s fathers at flash strengths of -0.119, 0.114, 0.4, 0.48, 0.6, 0.8 and 0.95 log cd.s.m^− 2^ (*p* < 0.05, see Supplementary Figure S2). Similar findings in ASD’s mothers, their time-to-peak of the b-wave were significantly slower than control individuals at flash strengths of − 0.119, 0.114 and 0.6 log cd.s.m^− 2^ (*p* < 0.01), whereas those of ASD’s siblings were slower than that of control group at − 0.119 log cd.s.m^− 2^ only (*p* < 0.05). The results indicated that their parents showed a longer time-to-peak for b-wave amplitudes than the control subjects. For the PhNR parameters of amplitudes or time, no significant differences were found at all the flash strengths on all the measures between the controls and ASD (*p* > 0.61), and amongst the other ASD family member groups (*p* > 0.07).

The age of participant was positively correlated with a-wave time-to-peak at high flash strength (0.114 and 1.204 log cd.s.m^− 2^, p-values = 0.016 and < 0.001 respectively, see Supplementary Table S4), with b-wave time-to-peak at all light strengths (p-values between 0.002 to < 0.001), and also with the time PhNR at minimum amplitude (Tmin) at 1.204 log cd.s.m^− 2^ (*p* = 0.035).

#### Relationship between motion coherence thresholds and ERG parameters

The iris colour index was not correlated with mean motion coherence thresholds (*N* = 110), *r*=− 0.166, *p* = 0.074). The motion coherence thresholds were correlated with a-wave time-to-peak at − 0.119 and 0.4 log cd.s.m^− 2^ both for BoW and WoB (p-values between 0.001 and 0.048 with r between − 0.32 and − 0.194, in Supplementary Table S4), as well as with b-wave time-to-peak at − 0.119, 0.114, 0.4, 0.6, 1.114 log cd.s.m^− 2^ mainly for WoB (p-values between 0.004 and 0.044 with r between 0.199 and 0.283). The parameters of PhNR amplitude (both at 72 min and Tmin) were negatively correlated with motion coherence thresholds (both for BoW and WoB) at 0.114 log cd.s.m^− 2^ whereas only with the thresholds for WoB at 1.204 log cd.s.m^− 2^ (p-values between 0.005 and 0.035 with r between − 0.207 and − 0.274). These findings have demonstrated the shorter a- and b-wave time-to-peak and the smaller PhNR amplitudes (both p72 and Tmin) required higher motion detection thresholds.

Comparisons between all the ERG parameters between normal and abnormal motion coherence thresholds showed multiple statistically significant differences with the a-wave time-to-peak (at − 0.119, 0.4 and 1.204 log cd.s.m^− 2^, p-values between 0.011 and 0.016, see Supplementary Table S5), a-wave amplitude (at 1.114 and 1.204 log cd.s.m^− 2^, p-values = 0.042 and 0.022 respectively), b-wave time-to-peak (at 0.114, 0.4 and 0.6 log cd.s.m^− 2^, p-values between 0.012 and 0.047), Tmin (at − 0.119 and 0.6 log cd.s.m^− 2^, p-values = 0.026 and 0.031 respectively), p72 (at 0.114 and 1.204 log cd.s.m^− 2^, p-values = 0.049 and 0.012 respectively), PhNR_Tmin at 1.204 log cd.s.m^− 2^, p-value = 0.04). The amplitudes of the a-wave and PhNR (both p72 and PhNR_Tmin) were significantly smaller in those with abnormal motion coherence compared to those with normal motion coherence detection. The a-wave time-to-peak of those with abnormal motion coherence were significantly shorter than those with normal detection thresholds, whereas the b-wave time-to-peak and Tmin were vice versa.

#### The effects of IQ, autism severity, comorbidities and medications on ERG parameters

There were no differences of all the ERG measures between those with or without comorbidities (all p-values > 0.053), and between those taking CNS medications (all p-values > 0.069) in the ASD families. Full-scale IQ score was positively correlated with a-wave time-to-peak (at 0.4 log cd.s.m^− 2^, *r* = 0.347, *p* = 0.03, see Supplementary Table S6) and b-wave time-to-peak (at 0.48 ISCEV, 0.8 and 1.204 log cd.s.m^− 2^, *r* = 0.378, *p* = 0.018; *r* = 0.359, *p* = 0.025 and *r* = 0.343, *p* = 0.032 respectively). ADOS score was positively correlated with b-wave amplitude at − 0.367 log cd.s.m^− 2^ (*r* = 0.346, *p* = 0.033), but negatively correlated with PhNR amplitudes (p72) at ISCEV flash strength (*r*=− 0.376, *p* = 0.022) and Tmin at 1.204 log cd.s.m^− 2^ (*r*=− 0.440, *p* = 0.006). Autism severity score was also negatively correlated with PhNR amplitudes (p72) at ISCEV flash strength (*r*=− 0.374, *p* = 0.019), PhNR at Tmin both at ISCEV flash strength (*r*=− 0.354, *p* = 0.029) and 1.204 log cd.s.m^− 2^ (*r*=− 0.412, *p* = 0.008).

### Bayesian analysis

We have performed Bayesian network analysis to examine the effects of ERG parameters or phenotypic variables on motion coherence thresholds. There were no effects from full-scale IQ [Bayes Factor (BF_10_) = 0.3, see Supplementary Table S7], ADOS total score (BF_10_ = 1.7), autism severity score (BF_10_ = 2.0) and iris index colour (BF_10_ = 0.7) on motion coherence thresholds. Since BF_10_ between 3 and 10 indicates moderate evidence for an effect, Bayesian t-tests showed evidence in favour of a moderate positive association between motion coherence threshold with participant’s age (BF_10_ = 8.2) and all the ERG parameters, such as a-wave time-to-peak (BF_10_ = 4.3 at 0.114 log cd.s.m^− 2^), a-wave amplitude (BF_10_ = 8.7 at 0.8 log cd.s.m^− 2^), b-wave time-to-peak (BF_10_ = 8.6 at ISCEV flash strength 0.48 log cd.s.m^− 2^), b-wave amplitude (BH_10_ = 8.8 at 0.4 log cd.s.m^− 2^), Tmin (BF_10_ = 8.7 at 0.4 log cd.s.m^− 2^), and p72 (BF_10_ = 8.6 at 0.4 log cd.s.m^− 2^) and PhNR_Tmin (BF_10_ = 8.6 at 0.95 and 1.114 log cd.s.m^− 2^). These results suggest participant’s age and all ERG parameters had effect on motion perception.

## Discussion

**Fig. 5:**
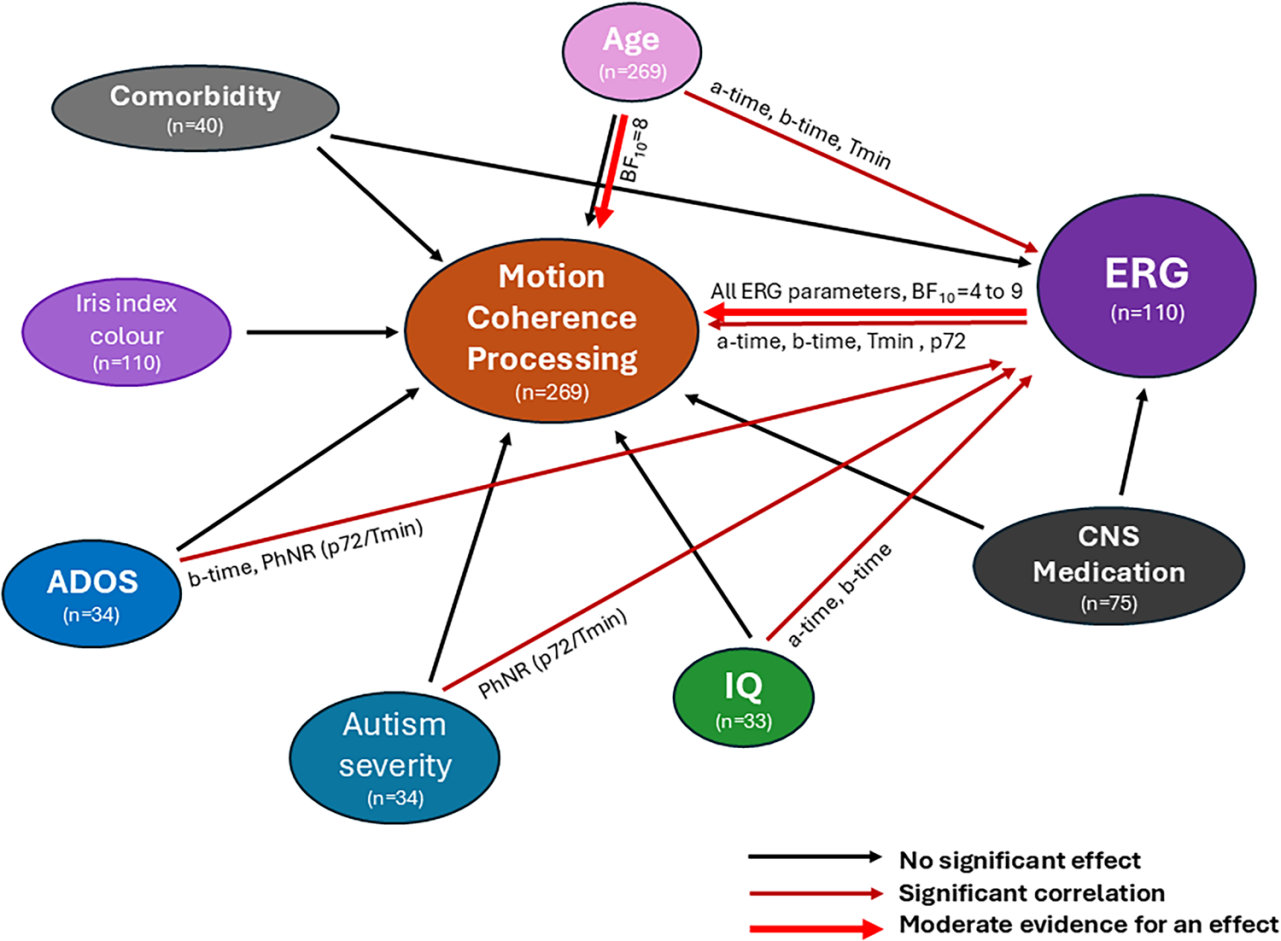
Figure 5 displays the relationship between the variables affecting global motion coherence thresholds in the ASD families and control participants. *CNS* = Central nervous system, *ADOS* = Autism Diagnostic Observation Schedule total score, *IQ* = Intelligence quotient, *ERG* = Electroretinogram, *a-wave time* = a-wave time-to-peak (ms), *b-wave time* = b-wave time-to-peak (ms), *Tmin* = the time PhNR at minimum amplitude (ms), *PhNR p72* = PhNR amplitude at t = 72ms (*u*V), *PhNR Tmin* = PhNR amplitude at Tmin (*u*V), *BF*_*10*_ = Bayes Factor (= 1 means no evidence (inconclusive); 3–10 means moderate evidence for effect). Black arrow means there was no statistical significance between variables. Maroon arrow indicates a statistically significant correlation between variables. Red thicker arrow indicates moderate evidence for an effect by Bayesian analysis.

This is the first report to show motion coherence deficits in ASD families was associated with retinal alterations which interacted with age, intellectual abilities and autism severity. Figure 5 summarises the relationship between the variables affecting the global motion coherence processing.

The findings of this study show that motion coherence thresholds are age-dependent in control individuals. Visual acuity is known to mature until up to six years of age^41^. Previous studies have reported that motion coherence thresholds reach adult-like levels between the ages of 10 and 14 years of age^42,43^, with a gradual decline with age^44^. In agreement, these results showed that older control individuals had higher motion coherence thresholds than younger participants at identifying the direction of global motion in a random-dot kinematogram, replicating previous findings^45–50^.

In the ASD family groups, 55% of the ASD participants had elevated motion coherence thresholds. This finding is in keeping with previous reports that have found ASD individuals to have significantly higher motion coherence thresholds than typically developed comparison groups^24,51–53^. However, researchers find that there is a substantial amount of individual variability, with approximately only 22–40% of individuals with autism showing elevated motion coherence thresholds^23,52,54^. Within the ASD families of our study, over 40% of both the ASD’s siblings and the ASD’s mothers had significantly higher motion coherence thresholds than the comparison control group (9.2%). These results show that many of the ASD probands, their siblings and mothers required more coherently moving dots for motion detection. These findings suggest that the phenotype of global motion perception deficits follow a familial pattern in the ASD families, predominantly expressed as a risk trait in the siblings and possibly from the ASD’s mothers in this population sample.

The intelligence, autism severity, comorbidities and central nervous system medications were not associated with the motion coherence deficits in the ASD families. A comprehensive meta-analysis encompassing 48 studies found that individuals with ASD exhibit a small but consistent deficit in global motion perception, and these deficits are independent of age or IQ^4^. The direct effects of CNS medications on global motion processing in ASD are not well-documented. A research study demonstrated a psychotropic medication, propranolol may affect functional connectivity in individuals with ASD, potentially influencing sensory processing pathways^55^. A study found that in schizophrenia patients, global motion deficits were not solely attributable to antipsychotic treatment^56^, while another study by Chen et al. (2011) reported that the deficits could be potentially modulated by antipsychotic medications^57^.

Altered retinal function has been reported in recent studies in ASD^28,35,36^. In this study, the reduction of LA-ERG a- and b-wave amplitudes in ASD probands compared with that of the control group replicated previous findings in this cohort. Similar attenuation of a- and b-wave amplitudes in ASD probands compared to their parents and siblings were also found. Realmuto et al. reported in 1989 that the dark-adapted ERG b-wave amplitudes were abnormal in probands and their first-degree relatives^39^. The early component of the ERG waveform is generated by the photoreceptors, horizontal cells and bipolar cells^58^. Their alterations in the initial visual processing to light in ASD probands may imply a different way of communication and interconnection between photoreceptors and bipolar cells. The delayed time-to-peak of the b-wave has also been found in ASD’s parents as well as in ASD’s siblings. This may be due to insensitive interactions in the neuronal circuits and synapses within the retina. The synaptic interaction is regulated by glutamate signalling between the cone and bipolar cells and the inhibitory GABA neurotransmitter from horizontal cells between the cone and horizontal cells^59^. GABAergic and glutaminergic pathway alter the synaptic transmissions, consequently leading to imbalance of excitation and inhibition of neurotransmission^60–62^. A UK ERG twin study demonstrates significant heritability on multiple ERG parameters, indicating the importance of genetic factors to the retinal electrophysiologic function^63^.

The synaptic communication associated with slower and smaller ERG amplitudes between the cone photoreceptor, ON-and OFF-bipolar cells and horizontal cells in ASD may in turn alter retinal ganglion cell (RGC) activity. In our results, there were no significant differences between the amplitudes of PhNR, a global RGC and glial generated signal, in ASD probands and controls, the same as previously reported in Constable et al. (2021) using different datasets^37^. Additionally, no differences in PhNR amplitudes were found between ASD probands and their family members. The correlation of participant age with the ERG parameters suggested that the older the participants the longer the time-to-peak of a- and b-waves, and the time to the PhNR. However, further investigations on the relationship between the ERG parameters and motion coherence thresholds showed significant correlations between them, and there were also significant differences in the ERG parameters between individuals with normal and abnormal motion coherence thresholds. Smaller amplitudes of the ERG parameters (mainly a-wave and PhNR) were found in those with abnormal motion detection thresholds. The results from Bayesian network analyses demonstrated a moderate effect from participant’s age and all ERG parameters on global motion perception deficits. These findings suggest associations between the alterations of the ERG measures with the motion detection thresholds in this cohort. Therefore, the motion processing deficits in the ASD probands and their family members may be due to an imbalance in neuronal transmission starting from retinal pathway during visual processing.

The PhNR has been established as an objective functional test for optic nerve and retinal diseases involving RGC injury^64^. Motion perception is understood to start in RGC that project to the lateral geniculate nucleus (LGN), in particular the magnocellular system^30,44,65,66^. The LGN then projects to neurons in the primary visual area (V1). Global motion is processed in the middle temporal area (MT/V5), which receives direct connections from V1 and indirect ones via V2 and V3^44,67^. Jure (2018) has found a direct relationship in the degree of compromise on peripheral vision secondary to dysfunctions on the magnocellular pathway and the degree of autism severity^68^. The motor perception deficits in ASD and family members may be affected by inherited traits in their genome, which are associated with this pathway. Further genetic study on these families may confirm this indication. It has been suggested that the effects of common genetic variations on cognitive functions are magnified by age, thus, increasing inter-individual differences^69,70^, and also that individual differences in motion perception are related to genetic variations^44^. This may explain why the older age group had higher motion coherence thresholds, possibly with more genetic variations over the years and age-related decline in visual perception^71–73^.

A primary limitation of this study is incomplete phenotypic data on cognitive measure (IQ scores) and autism severity scores (ADOS score) to test all the participants in this study that might have introduce bias in the interpretation of the results. Also, larger sample sizes for the parental groups both in ASD and control families could provide clearer observations for the investigation on retinal function and motion perception. Recruitment from a wider community across the country for the control group may reduce selection bias. Another limitation is the lack of genetic information for the ASD family members. Future research could explore the relationship between genetic factors and motion perception deficits in the ASD families.

## Conclusion

The coherence motion detection thresholds were abnormally high in ASD probands, their siblings and mothers. Significant attenuation of ERG a-wave and b-wave amplitudes and increase of b-wave time-to-peak were found in ASD probands compared with those of control subjects replicating the findings in Constable et al. (2020). The altered retinal function was associated with the age, intelligence and autism severity of the ASD family members. Elevated motion coherences in ASD were associated with altered retinal signalling measured with LA ERGs. The mechanism of the motion coherent deficits in individuals with ASD and their family members may start from the slightly altered retinal functioning. The findings imply altered retinal function and motion coherence deficits are a potential inherited risk factor for ASD and further study with a genetic study could give insight on the findings. Since the results have shown that global motion processing relies on distinct neural pathways, future studies could explore whether the deficits are specific to global motion processing or also involve local motion processing.

## Methods

The study was conducted across two sites based in London (UK) and Adelaide (Australia). Local Human Research Authority approval was received to conduct the study at Great Ormond Street Hospital by the South East Scotland Research Ethics Committee in the UK (Approval Code:18/SS/0008) and at the Flinders University by the Women’s and Children’s Health Network Human Research Ethics Committee in Australia (Approval Code: 7180). Written informed consent was obtained from the parent/guardian or the individual participant if older than 16 years old. The project received local ethical approval for the study protocols and was conducted in accordance with the Declaration of Helsinki.

### Participants

All participants in this study had normal or corrected-to-normal visual acuity with the exclusion criteria of previous ocular surgery, strabismus and inherited retinal diseases. All participants had acuity > 6/6 in each eye and had no other eye conditions nor taking any medications for correcting retinal dysfunction.

#### ASD group

All ASD participants met DSM-IV or DSM-5 (Diagnostic and Statistical Manual of Mental Disorders) criteria based on assessment with ADOS or ADOS-2 (Autism Diagnostic Observation Schedule) and the developmental, dimensional and diagnostic interview (3Di)^74^ assessed by paediatric psychiatrists or clinical psychologists in the social communication disorder clinics at the Great Ormond Street Hospital in the UK or local Child and Adolescent Mental Health clinics. The exclusion criteria for recruitment was whether a participant had: any history of ocular disease or strabismus; a congenital syndrome such as Fragile-X, Downs or Rett’s; any history of brain trauma or pathology; a history of epileptic seizures in the last year; with full scale IQ < 65 and/or was unable to follow simple verbal instructions. Autism Severity Scores were calculated using the methods of Gotham et al. (2009)^75^.

43 participants with ASD were recruited, of whom 74.4% were males. The mean age(SD) and range were 14.8(4.6) and 6–27 years. The ADOS total scores and severity scores were 11.8(4.7) and 6.8(1.8) respectively. The ASD group was categorised as high functioning with a mean full-scale IQ 98.5(19.4). Some of these ASD individuals had also been diagnosed with comorbidities: 13 had ADHD (of which, 1 also had ODD and language disorder), 1 had OCD, 1 had OCD and Dyslexia, and 1 had myalgic encephalomyelitis. The ASD participants required medications: 4 were on ASD dopamine re-uptake inhibitors, 3 on selective serotonin reuptake inhibitors (SSRI), 3 on melatonin at night, 2 on antihistamines/asthma inhalers, 1 was taking vitamin supplements, 1 was on an alpha-2 agonist or an asthma inhaler, or a proton pump inhibitor, and 1 had taken antiepileptic medication.

#### ASD family group

Out of 43 ASD participants, 12 of the ASD families including parents and siblings took part in the testing. 6 fathers were included with an age mean of 48.7(3.5) years and age range of 44–53, and 12 mothers aged from 39 to 58 with a mean of 49.6(5.7) were recruited. 20 siblings of the ASD probands also participated, with an age mean of 15.8(4.6), ranging from 8 to 27 years and 30% of them were male.

Some parents had various types of diagnoses - in the maternal group, 2 had depression; 2 had diabetes (1 also had asthma); 1 had post-thyroidectomy and severe migraine; 1 had asthma; and 1 had mental illness and required psychiatric medications. For the paternal group one had an orthopaedic diagnosis and required anti-inflammatory and analgesic medications. All these parents had taken medications before ERG testing. The siblings of the ASD group included 4 with dyslexia (of which, 1 also had ADHD), but were unmedicated. One had OCD and had taken medications before testing and one other was using a SSRI medication but had no neurodevelopmental condition.

#### Control group

A total of 194 controls were recruited from local schools and colleagues with no first-degree family member with an ASD diagnosis and had no mental health condition or developmental delay. The control group’s age was 24.2(16.1) and ranged between 4 and 70 years. Not all participants underwent electrophysiology testing. 29 control subjects acted as controls for the LA3 ERG comparison. The ERG group had an age range from 6 to 24 years with a mean age of 15.3(4.6) years. Amongst these individuals, there were 10 families tested. 1 mother of a control child took post-thyroidectomy medications before the testing, and 1 child had just been diagnosed with diabetes. All control participants had no medications.

A recruitment flowchart in Supplementary Figure S3 displays all the numbers of cases taken part in the motion coherence and electroretinogram tests in different age groups.

### Motion coherence test

A classical random dot setup was applied for assessing the participant’s global coherent motion perception with the LumiTrack™^76^. Participants sat in front of a laptop computer at a distance of 0.5 m and were shown randomly-moving dots on the screen (noise dots) with a fraction of them moving coherently in one direction (signal dots). A stimulus based on Brownian motion was chosen as it is robust to changes in contrast, speed, aperture size, as well as spatial displacement and the temporal displacement of dots^77^. Participants were asked to indicate the direction of the moving signal dots towards a schematic house or tree using either a keypress, by pointing or by verbalising the direction of motion.

The task was a Two-Alternative-Forced Choice (2AFC) scenario. As an example, the participant began the test at 100% coherence with all of the 1081 signal dots moving in one random coherent direction. Following a correct answer, the coherence level was lowered to 50%, so that half the dots remained as signal dots while the other half became noise dots. Another correct response would render the signal dots at 25% and the noise dots at 75%. The difficulty of the task increased and decreased depending on the participant’s answers, by changing the signal/noise ratio. The test continued following the underlying staircase algorithm until ten reversals were reached. The resulting value, as a threshold, is determined as the percentage of total signal dots, using a 2AFC 3 down and 1 up adaptive staircase with 79.4% convergence, which was directly translatable into an absolute measure of the amount, or the percentage, of signal dots required to perceive a global coherent motion in one direction. The staircase was terminated after 10 reversals and the resulting threshold value was calculated by taking the average of the last eight reversal values. The percentage thresholds 25% or above for each participant were regarded as abnormal motion detection thresholds^76^.

**Fig. 6:**
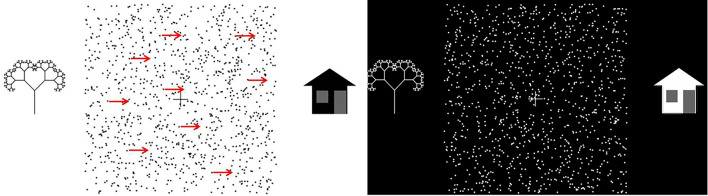
Figure 6 shows the coherent motion tests with moving black dots on a white background (left) and white dots on black background (right). Participants would indicate which direction the dots were coherently moving: either towards the tree on the left or the house on the right using a keypress, or pointing or verbalising the direction of motion towards the ‘tree’ or ‘house’.

The task was performed with black dots on a white background (BoW) and with white dots on a black background (WoB). The motion coherence threshold of each participant was represented by taking average of BoW and WoB for analysis. See Fig. 6 for example of the task and 2 videos (in supplementary audio-visual file: Motion coherence_WoB.mp4 and Motion coherence_BoW.mp4) shows the coherently moving signal dots with the randomly moving dots.

### Electroretinogram

The ERG was recorded following the guidelines of the International Society for Clinical Electrophysiology of Vision (ISCEV) standard^31^. The ERG measures of both eyes included the a-, b-wave amplitudes and time-to-peak, and the PhNR (including p72, Tmin, BT, p-ratio and w-ratio) parameters at all the nine flash strengths and the ISCEV standard flash. The details of these ERG parameters are described in Supplementary Note S1. This study involved the LA-ERG recordings which were part of a multicentre project previously published^36–38,78^.

### Statistical analysis

The motion coherence test thresholds from BoW and WoB stimuli were averaged for each participant and compared amongst the groups using t-test or one-way ANOVA. The ERG measures of 29 control individuals were compared with those of the 12 ASD family member groups. All the ERG parameters at all the nine flash strengths and the ISCEV standard flash were compared amongst the ASD family members and control groups using a general linear model and Bonferroni corrections applied to account for Post Hoc tests. Correlations and Bayesian analyses were employed to investigate the relationship between all the ERG parameters and motion coherence thresholds. The analysis was run in SPSS software (Version 29) and Origin 2022b. A two-sided p-value less than 0.05 was considered as statistically significant.

## Electronic supplementary material

Below is the link to the electronic supplementary material.

Supplementary Material 1

Supplementary Material 2

Supplementary Material 3

## Data Availability

The datasets used and/or analysed during the current study available from the corresponding author on reasonable request.
