# Correction: Global motion coherent deficits in individuals with autism spectrum disorder and their family members are associated with retinal function

**DOI:** 10.1038/s41598-025-20021-w

**Published:** 2025-09-24

**Authors:** Irene O. Lee, Dennis M. Fritsch, Maximilian Kerz, Jane C. Sowden, Paul A. Constable, David H. Skuse, Dorothy A. Thompson

**Affiliations:** 1 Behavioural and Brain Sciences Unit, Population Policy and Practice Programme, Great Ormond Street Institute of Child Health, University College London, London, UK; 2 Oceano Azul Foundation and Katapult Ocean, Baden Württemberg, Germany; 3 Cherry Health, Calgary, Canada; 4 Great Ormond Street Institute of Child Health, University College London and Great Ormond Street Hospital NIHR Biomedical Research Centre, London, UK; 5 College of Nursing and Health Sciences, Caring Futures Institute, Flinders University, Adelaide, Australia; 6 Great Ormond Street Institute of Child Health, University College London, London, UK; 7 Clinical and Academic Department of Ophthalmology, Great Ormond Street Hospital for Children NHS Trust, London, UK

Correction to: *Scientific Reports* 10.1038/s41598-025-11789-y, published online 02 August 2025

The original version of this Article contained errors in the Figures.

In Figure 1, the CTL Exponential Equation was incorrect. As a result, the curve in the graph has been recalculated. Also, in the yellow box in the figure, “ < 25% threshold” has now been corrected to “ ≥ 25% threshold”.

In Figure 2, part of the legend was omitted and was erroneously added to the Results section, under the subheading ‘Motion coherence test’. As a result,

“Box plots to show the elevated percentage thresholds of motion coherence of control group and the ASD family members. The percentage motion coherence thresholds on the y-axis were the percentage of the total signal dots and were average of positive and negative contrast WoB and BoW tests from white dots on a black background and black dots on a white background respectively.”

now reads:

“Box plots to show the elevated percentage thresholds of motion coherence of control group and the ASD family members. The percentage motion coherence thresholds on the y-axis were the percentage of the total signal dots and were average of positive and negative contrast WoB and BoW tests from white dots on a black background and black dots on a white background respectively. Figure 2a shows % coherence thresholds of Control in each age group; Fig. 2b shows % coherence thresholds of ASD family and control groups; Fig. 2c shows % coherence thresholds of each age group in different family member groups. CTL=Control; Fathers=ASD’s fathers; Mothers=ASD’s mother; Sib=ASD’s sibling. % Motion coherence thresholds≥25% were considered abnormal. ***p<0.01. Median line and mean (box shape) of each group are shown within each box plot.”

In Figure 3a, the brackets for the groups “Children & young persons” and “Children & young persons’ parents” were omitted.

In Figure 5, part of the legend was omitted and was erroneously added to the Discussions section. As a result,

“*CNS* = Central nervous system, *ADOS* = Autism Diagnostic Observation Schedule total score, *IQ* = Intelligence quotient, *ERG* = Electroretinogram, *a-wave time* = a-wave time-to-peak (ms), *b-wave time* = b-wave time-to-peak (ms), *Tmin* = the time PhNR at minimum amplitude (ms), *PhNR p72* = PhNR amplitude at t = 72ms (*u*V), *PhNR Tmin* = PhNR amplitude at Tmin (*u*V), *BF*_*10*_ = Bayes Factor (= 1 means no evidence (inconclusive); 3–10 means moderate evidence for effect). Black arrow means there was no statistical significance between variables. Maroon arrow indicates a statistically significant correlation between variables. Red thicker arrow indicates moderate evidence for an effect by Bayesian analysis.”

now reads:

“Figure 5 displays the relationship between the variables affecting global motion coherence thresholds in the ASD families and control participants. *CNS* = Central nervous system, *ADOS* = Autism Diagnostic Observation Schedule total score, *IQ* = Intelligence quotient, *ERG* = Electroretinogram, *a-wave time* = a-wave time-to-peak (ms), *b-wave time* = b-wave time-to-peak (ms), *Tmin* = the time PhNR at minimum amplitude (ms), *PhNR p72* = PhNR amplitude at t = 72ms (*u*V), *PhNR Tmin* = PhNR amplitude at Tmin (*u*V), *BF*_*10*_ = Bayes Factor (= 1 means no evidence (inconclusive); 3–10 means moderate evidence for effect). Black arrow means there was no statistical significance between variables. Maroon arrow indicates a statistically significant correlation between variables. Red thicker arrow indicates moderate evidence for an effect by Bayesian analysis.”

The original Figs. 1 and 3 and their accompanying legends appear below.

**Fig. 1:**
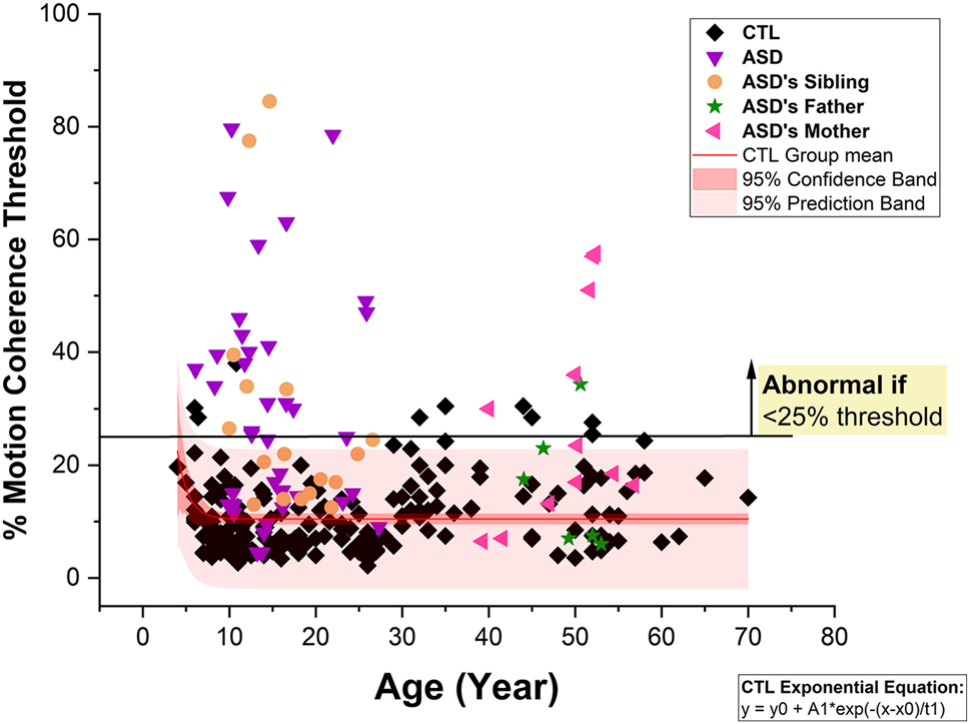
Percentage thresholds of motion coherence against age of the control individuals and autism family members. CTL = control individuals, ASD = autism spectrum disorder. Total Signal Dots were 1081. The red straight line presents the group mean, 95% confidence interval in dark red band and 95% prediction interval in light red band.

**Fig. 3:**
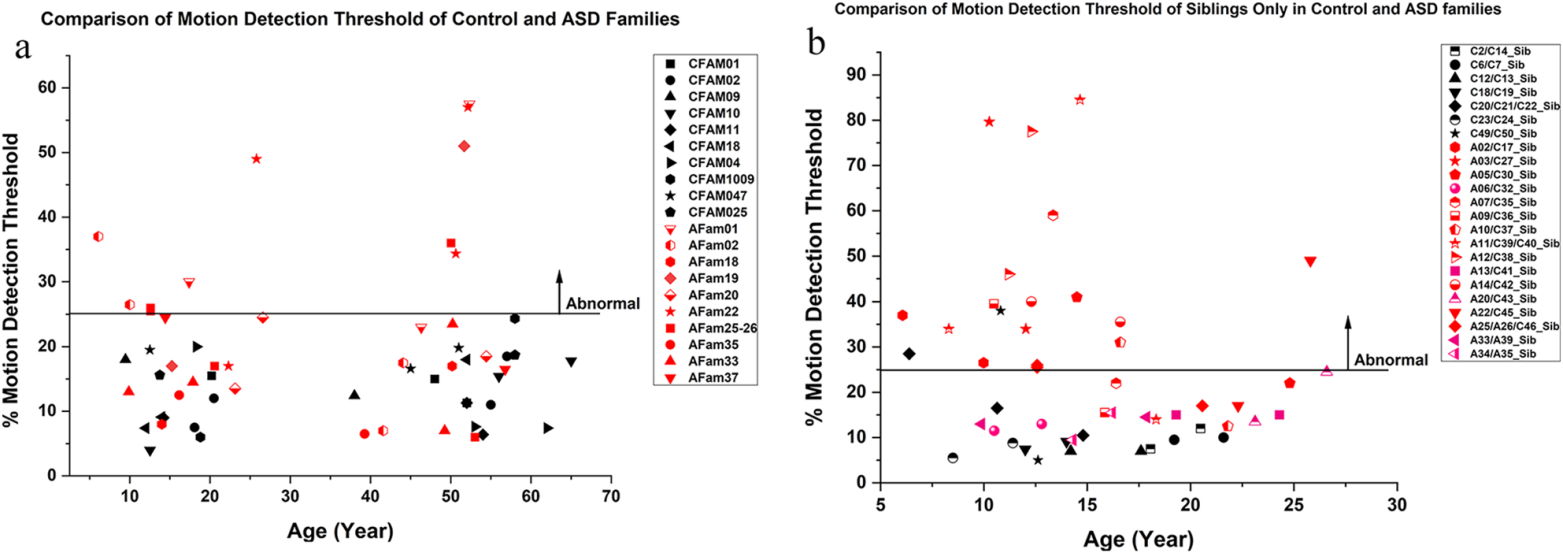
Comparison of the motion coherence thresholds between Control and ASD family members. Each family has the same shape and colour. (**a**) Comparison of the percentage motion detection thresholds of 10 Control Families (CFam) and 10 Autism Families (AFam), including their parents and siblings. (**b**) Comparison of the percentage motion detection thresholds of siblings only. Black signs represent control siblings from 7 families and red are from 16 ASD probands’ siblings. The codes start with C or A represents those with or without ASD respectively.

The original Article has been corrected.

