# Supplementary material for: Global motion coherent deficits in individuals with autism spectrum disorder and their family members are associated with retinal function
Source: Sci Rep. 2025 Aug 2;15:28249. doi: 10.1038/s41598-025-11789-y (PMC12318104; doi:10.1038/s41598-025-11789-y)
Supplement: Supplementary file 3 — Supplementary Material 3 [file 41598_2025_11789_MOESM3_ESM.docx]

**Supplementary Information**

**Global motion coherent deficits in individuals with autism spectrum disorder and their family members are associated with retinal function**

Irene O Lee^1*^, Dennis M Fritsch^2^, Maximilian Kerz^3^, Jane C Sowden^4^, Paul A Constable^5^, David H Skuse^1^, Dorothy A Thompson^6, 7^

^1^ Behavioural and Brain Sciences Unit, Population Policy and Practice Programme, Great Ormond Street Institute of Child Health, University College London, London, UK,

^2^ Oceano Azul Foundation and Katapult Ocean, Baden Württemberg, Germany,

^3^ Cherry Health, Calgary, Canada,

^4^ Great Ormond Street Institute of Child Health, University College London and Great Ormond Street Hospital NIHR Biomedical Research Centre, London, UK,

^5^ College of Nursing and Health Sciences, Caring Futures Institute, Flinders University, Adelaide, Australia,

^6^ Great Ormond Street Institute of Child Health, University College London, London, UK,

^7^ Clinical and Academic Department of Ophthalmology, Great Ormond Street Hospital for Children NHS Trust, London, UK.

**Contents**

[**Supplementary Table S1.** The percentage motion coherence thresholds of both motion tests that are white dots on a black background (WoB) and black dots on a white background (BoW). 3](#_Toc196807132)

[**Supplementary Table S2.** Gender differences of Motion coherence thresholds in each age group of Control and ASD family member groups. 4](#_Toc196807133)

[**Supplementary Table S3.** The effects of IQ, autism severity, comorbidities and medications on motion coherence deficits in ASD families. 5](#_Toc196807135)

[**Supplementary Figure S1.** a-wave amplitudes of ASD family members and control individuals. 6](#_Toc196807136)

[**Supplementary Figure S2.** Time-to-peak of b-waves of the ASD family member and control groups. 7](#_Toc196807137)

[**Supplementary Table S4.** Correlations between the ERG parameters and age of participant, and motion coherence thresholds at each light strength. 8](#_Toc196807138)

[**Supplementary Table S5.** Comparisons of the ERG parameters between normal and abnormal motion coherence thresholds at each light strength. 14](#_Toc196807139)

[**Supplementary Table S6.** Correlations of full-scale IQ, ADOS and autism severity scores with ERG parameters in ASD families. 21](#_Toc196807140)

[**Supplementary Table S7.** Bayesian analysis to compare the ERG parameters between normal and abnormal motion coherence thresholds at each phenotypic variable and light strength. 22](#_Toc196807141)

[**Supplementary Figure S3.** Recruitment Flowchart. 27](#_Toc196807142)

[**Supplementary Note S1.** ERG parameters 28](#_Toc196807143)

[**References** 29](#_Toc196807144)

# **Supplementary Table S1.** The percentage motion coherence thresholds of both motion tests that are white dots on a black background (WoB) and black dots on a white background (BoW).

|  |  | **% Motion Coherence Thresholds** | | | | | | |  |
| --- | --- | --- | --- | --- | --- | --- | --- | --- | --- |
|  | **Age group** |  | **BoW** | |  | **WoB** | | **BoW cf WoB** | |
|  | **(year)** | **N** | **mean±SD** | **Median** | **N** | **mean±SD** | **Median** | ***p-value*** | |
| **Control** | ≤16 | 66 | 9.9±7.1 | 7.4 | 71 | 10.7±9.8 | 8.5 | 0.60 | |
|  | 17-27 | 41 | 8.0±4.4 | 7.0 | 41 | 7.9±4.0 | 7.5 | 0.92 | |
|  | 28-70 | 61 | 15.7±8.2 | 14.0 | 60 | 14.2±7.5 | 13.9 | 0.32 | |
|  | Total* | 168 | 11.9±8.1 | 8.9 | 172 | 11.2±8.3 | 9.1 | 0.75 | |
| **ASD** | ≤16 | 31 | 28.7±19.8 | 25.5 | 31 | 28.7±19.8 | 25.5 | 0.72 | |
|  | 17-27 | 9 | 31.3±22.9 | 25.0 | 9 | 31.3±22.9 | 25.0 | 0.77 | |
|  | Total | 40 | 29.2±20.2 | 25.3 | 40 | 29.2±20.2 | 25.3 | 0.88 | |
| **ASD’s Sibling** | ≤16 | 10 | 36.5±20.1 | 25.0 | 10 | 36.5±20.1 | 25.0 | 0.99 | |
|  | 16-27 | 7 | 22±16.7 | 22.3 | 7 | 22±16.7 | 22.3 | 0.96 | |
|  | Total | 17 | 28.7±19.0 | 22.0 | 17 | 28.7±19 | 22.0 | 0.77 | |
| **ASD’s Parents** | ASD’s Father | 6 | 15.9±11.3 | 12.5 | 6 | 15.9±11.3 | 12.5 | 0.99 | |
|  | ASD’s Mother | 12 | 25.1±16.8 | 18.5 | 12 | 25.1±16.8 | 18.5 | 0.94 | |
|  | Total | 18 | 23.5±16.7 | 18.0 | 18 | 23.8±19.2 | 17.5 | 0.95 | |
|  |  |  |  |  |  |  |  |  | |

N=Number of cases; SD=Standard deviation; BoW cf WoB= t-test was performed to compare the motion coherence thresholds between BoW and WoB tests. *In the control group, while there were 194 participants in total, some only took either BoW or WoB test and the remains had taken both tests.

# **Supplementary Table S2.** Gender differences of Motion coherence thresholds in each age group of Control and ASD family member groups.

|  |  | **% Motion Coherence Thresholds** | | | | | | |  | |
| --- | --- | --- | --- | --- | --- | --- | --- | --- | --- | --- |
|  |  | **Male** | | | **Female** | | | |  | |
|  | **Age Group**  **(year)** | **N** | **mean±SD** | **median** | **N** | **mean±SD** | **median** | ***p-value*** | |  |
| **CTL** | ≤16 | 43 | 9.3±6.6 | 7.4 | 42 | 10.4±6.2 | 9.1 | 0.46 | |  |
|  | 17-27 | 21 | 6.8±2.0 | 6.1 | 23 | 8.9±4.2 | 7.4 | 0.07 | |  |
|  | >28 | 27 | 12.1±7.1 | 11.3 | 38 | 16.0±6.3 | 15.0 | 0.03 | |  |
|  | Total | 91 | 9.6±6.4 | 7.4 | 103 | 12.1±6.6 | 11.0 | 0.19 | |  |
| **ASD** | ≤16 | 23 | 28.0±19.0 | 25.5 | 8 | 32.3±16.6 | 35.3 | 0.56 | |  |
|  | 17-27 | 6 | 24.9±21.5 | 15.0 | 3 | 18.2±5.9 | 15.0 | 0.25 | |  |
|  | Total | 29 | 28.4±19.6 | 25.0 | 11 | 28.4±15.6 | 25.0 | 0.88 | |  |
| **Sib** | ≤16 | 2 | 23.6±4.2 | 23.6 | 8 | 39.8±27.2 | 33.8 | 0.45 | |  |
|  | 17-27 | 2 | 23.3±1.8 | 23.3 | 5 | 15.2±2.1 | 15 | 0.01 | |  |
|  | Total | 4 | 23.4±2.6 | 23.3 | 13 | 24.3±24.3 | 17.5 | 0.59 | |  |
| **Parents** |  | 6 | 15.9±11.3 | 12.5 | 12 | 27.8±16.8 | 18.5 | 0.17 | |  |

CTL=Control; Sib=ASD’s sibling; N= number of subjects; SD= standard deviation.

# **Supplementary Table S3.** The effects of IQ, autism severity, comorbidities and medications on motion coherence deficits in ASD families.

Table S3a. Correlations of full-scale IQ, ADOS and autism severity scores with motion coherence threshold.

| **Phenotype** |  | **% Motion Threshold** |
| --- | --- | --- |
| **Full Scale IQ score** | Pearson Correlation (N) | -0.256 (33) |
|  | p-value | 0.151 |
| **ADOS score** | Pearson Correlation (N) | -0.016 (34) |
|  | p-value | 0.928 |
| **Autism Severity score** | Pearson Correlation (N) | -0.02 (34) |
|  | p-value | 0.912 |

IQ=Intellectual quotient; ADOS Total= Autism Diagnostic Observation Schedule total score; N=number of cases.

Table S3b. The effects of comorbidities and central nervous system medications on motion coherence deficits.

|  | **Number of cases** | **Normal MC** | **Abnormal MC** | **χ^2^/ PHI** | ***p-value*** |
| --- | --- | --- | --- | --- | --- |
| ASD Proband | No comorbidities | 11 (50%) | 11 (50%) | 0.494/ 0.111 | 0.482 |
|  | Has comorbidities | 7 (37%) | 11 (63%) |  |  |
| Took Medicine before testing* | No | 29 (54%) | 25 (46%) | 0.712/ 0.097 | 0.399 |
|  | Yes | 9 (43%) | 12 (57%) |  |  |
|  |  |  |  |  |  |

Note: *participants include ASD probands, ASD’s siblings and ASD’s parents; MC= motion coherence thresholds.

**Supplementary Figure S1.** a-wave amplitudes of ASD family members and control individuals. Multiple comparisons by Bonferroni tests were performed between ASD and ASD’s mothers, as well as between ASD and control group, *****p<0.05. Means and standard error bars are presented at each data point. Sib=ASD’s sibling; CTL=Control.


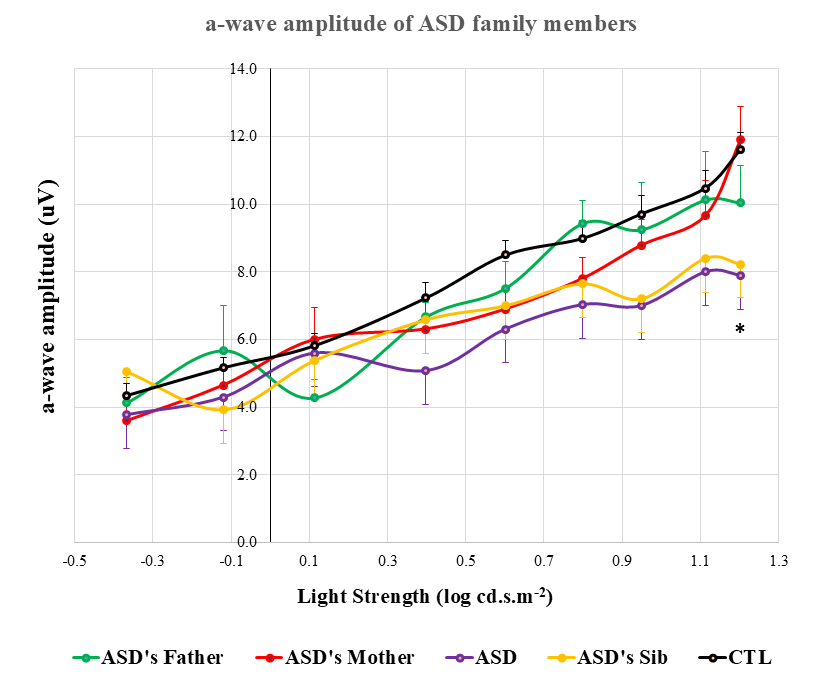


**Supplementary Figure S2.** Time-to-peak of b-waves of the ASD family member and control groups.

Multiple comparisons between ASD family member and control groups by Bonferroni tests were applied, *p<0.05; ǂp<0.01. Mean and standard error bars are presented in each column. Sib=ASD’s sibling; CTL=Control.


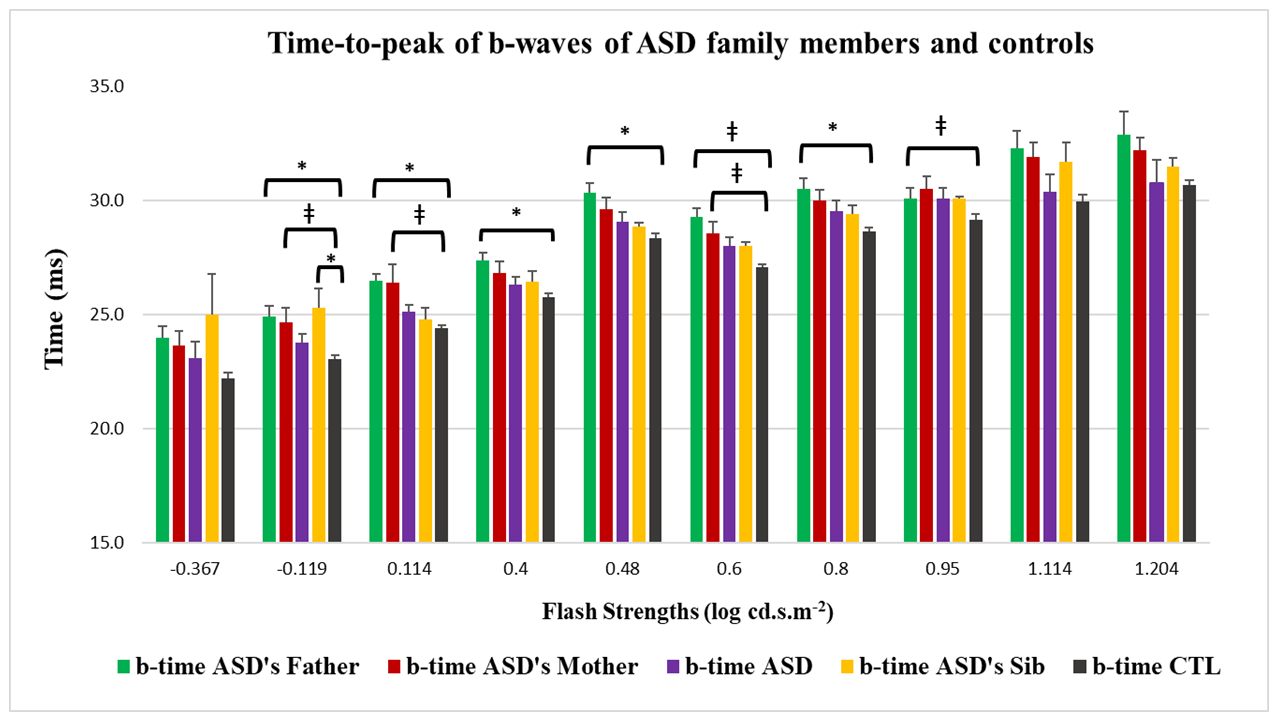


**Supplementary Table S4.** Correlations between the ERG parameters and age of participant, and motion coherence thresholds at each light strength.

The numbers in the left column presents the light strength of the flash in log cd.s.m^-2^; Sig.=statistically significant; N= number of subjects; BoW=motion detection thresholds for black dots on white background (%); WoB= motion detection thresholds for white dots on black background (%); Mean=mean of both motion detection thresholds for BoW and WoB (%); time=time-to-peak (ms); amp=amplitude (*u*V); p72 =PhNR amplitude at t=72ms (*u*V); Tmin= the time PhNR at minimum amplitude (ms); PhNR_Tmin= PhNR amplitude at Tmin (*u*V). ISCEV= International Society for Clinical Electrophysiology of Vision. Age=age of participant (year).

**The time-to-peak and amplitude of the a-wave and b-wave:**

| **ERG parameter** |  | **Age (year)** | **BoW (%)** | **WoB (%)** | **Mean (%)** |
| --- | --- | --- | --- | --- | --- |
| a-wave -0.367time (ms) | Pearson Correlation | -0.115 | -0.011 | -0.126 | -0.078 |
|  | Sig. (2-tailed) | 0.234 | 0.909 | 0.206 | 0.430 |
|  | N | 108 | 104 | 102 | 105 |
| a-wave -0.119time (ms) | Pearson Correlation | 0.021 | -0.194 | -0.244 | -0.221 |
|  | Sig. (2-tailed) | 0.825 | 0.047 | 0.013 | 0.023 |
|  | N | 111 | 105 | 103 | 106 |
| a-wave 0.114time (ms) | Pearson Correlation | -0.049 | -0.091 | -0.184 | -0.148 |
|  | Sig. (2-tailed) | 0.613 | 0.356 | 0.064 | 0.131 |
|  | N | 107 | 104 | 102 | 105 |
| a-wave 0.4time (ms) | Pearson Correlation | -0.147 | -0.194 | -0.32 | -0.279 |
|  | Sig. (2-tailed) | 0.125 | 0.048 | 0.001 | 0.004 |
|  | N | 110 | 105 | 103 | 106 |
| a-wave 0.48time (ms) | Pearson Correlation | -0.019 | 0.050 | -0.138 | -0.052 |
|  | Sig. (2-tailed) | 0.849 | 0.616 | 0.165 | 0.594 |
|  | N | 109 | 105 | 103 | 106 |
| a-wave 0.6time (ms) | Pearson Correlation | 0.064 | -0.049 | -0.162 | -0.117 |
|  | Sig. (2-tailed) | 0.504 | 0.618 | 0.102 | 0.232 |
|  | N | 110 | 105 | 103 | 106 |
| a-wave 0.8time (ms) | Pearson Correlation | 0.108 | 0.016 | 0.050 | 0.040 |
|  | Sig. (2-tailed) | 0.263 | 0.869 | 0.618 | 0.681 |
|  | N | 110 | 105 | 103 | 106 |
| a-wave 0.95time (ms) | Pearson Correlation | 0.094 | 0.057 | 0.011 | 0.039 |
|  | Sig. (2-tailed) | 0.335 | 0.562 | 0.916 | 0.691 |
|  | N | 108 | 104 | 102 | 105 |
| a-wave 1.114time (ms) | Pearson Correlation | 0.231 | 0.044 | 0.059 | 0.057 |
|  | Sig. (2-tailed) | 0.016 | 0.658 | 0.556 | 0.561 |
|  | N | 109 | 105 | 103 | 106 |
| a-wave 1.204time (ms) | Pearson Correlation | 0.360 | -0.071 | -0.104 | -0.091 |
|  | Sig. (2-tailed) | <0.001 | 0.473 | 0.296 | 0.355 |
|  | N | 110 | 105 | 103 | 106 |
| a-wave -0.367amp (*u*V) | Pearson Correlation | -0.119 | -0.032 | -0.059 | -0.053 |
|  | Sig. (2-tailed) | 0.221 | 0.751 | 0.559 | 0.588 |
|  | N | 108 | 104 | 102 | 105 |
| a-wave -0.119amp (*u*V) | Pearson Correlation | -0.066 | -0.038 | -0.159 | -0.112 |
|  | Sig. (2-tailed) | 0.492 | 0.698 | 0.109 | 0.254 |
|  | N | 111 | 105 | 103 | 106 |
| a-wave 0.114amp (*u*V) | Pearson Correlation | -0.115 | -0.017 | -0.075 | -0.063 |
|  | Sig. (2-tailed) | 0.239 | 0.861 | 0.454 | 0.526 |
|  | N | 107 | 104 | 102 | 105 |
| a-wave 0.4amp (*u*V) | Pearson Correlation | -0.047 | -0.037 | -0.152 | -0.115 |
|  | Sig. (2-tailed) | 0.623 | 0.705 | 0.125 | 0.241 |
|  | N | 110 | 105 | 103 | 106 |
| a-wave 0.48amp (ISCEV) (*u*V) | Pearson Correlation | -0.098 | 0.064 | -0.028 | -0.003 |
|  | Sig. (2-tailed) | 0.309 | 0.515 | 0.779 | 0.977 |
|  | N | 109 | 105 | 103 | 106 |
| a-wave 0.6amp (*u*V) | Pearson Correlation | -0.172 | 0.070 | 0.023 | 0.043 |
|  | Sig. (2-tailed) | 0.073 | 0.479 | 0.820 | 0.658 |
|  | N | 110 | 105 | 103 | 106 |
| a-wave 0.8amp (*u*V) | Pearson Correlation | -0.032 | -0.001 | -0.118 | -0.067 |
|  | Sig. (2-tailed) | 0.743 | 0.994 | 0.234 | 0.495 |
|  | N | 110 | 105 | 103 | 106 |
| a-wave 0.95amp (*u*V) | Pearson Correlation | -0.11 | -0.110 | -0.242 | -0.194 |
|  | Sig. (2-tailed) | 0.258 | 0.266 | 0.014 | 0.047 |
|  | N | 108 | 104 | 102 | 105 |
| a-wave 1.114amp (*u*V) | Pearson Correlation | -0.035 | -0.007 | -0.118 | -0.076 |
|  | Sig. (2-tailed) | 0.717 | 0.945 | 0.235 | 0.439 |
|  | N | 109 | 105 | 103 | 106 |
| a-wave 1.204amp (*u*V) | Pearson Correlation | 0.054 | -0.087 | -0.188 | -0.156 |
|  | Sig. (2-tailed) | 0.579 | 0.380 | 0.058 | 0.109 |
|  | N | 110 | 105 | 103 | 106 |
| b-wave -0.367time (ms) | Pearson Correlation | 0.295 | 0.050 | 0.110 | 0.093 |
|  | Sig. (2-tailed) | 0.002 | 0.611 | 0.273 | 0.347 |
|  | N | 109 | 104 | 102 | 105 |
| b-wave -0.119time (ms) | Pearson Correlation | 0.377 | 0.112 | 0.233 | 0.196 |
|  | Sig. (2-tailed) | <0.001 | 0.257 | 0.018 | 0.044 |
|  | N | 111 | 105 | 103 | 106 |
| b-wave 0.114time (ms) | Pearson Correlation | 0.510 | 0.156 | 0.279 | 0.239 |
|  | Sig. (2-tailed) | <0.001 | 0.114 | 0.004 | 0.014 |
|  | N | 108 | 104 | 102 | 105 |
| b-wave 0.4time (ms) | Pearson Correlation | 0.307 | 0.122 | 0.283 | 0.227 |
|  | Sig. (2-tailed) | 0.001 | 0.215 | 0.004 | 0.019 |
|  | N | 111 | 105 | 103 | 106 |
| b-wave 0.48time (ms) (ISCEV) | Pearson Correlation | 0.452 | -0.042 | 0.075 | 0.032 |
|  | Sig. (2-tailed) | <0.001 | 0.672 | 0.450 | 0.747 |
|  | N | 109 | 105 | 103 | 106 |
| b-wave 0.6time (ms) | Pearson Correlation | 0.436 | 0.120 | 0.259 | 0.213 |
|  | Sig. (2-tailed) | <0.001 | 0.225 | 0.009 | 0.030 |
|  | N | 110 | 104 | 102 | 105 |
| b-wave 0.8time (ms) | Pearson Correlation | 0.403 | 0.038 | 0.169 | 0.120 |
|  | Sig. (2-tailed) | <0.001 | 0.701 | 0.088 | 0.219 |
|  | N | 110 | 105 | 103 | 106 |
| b-wave 0.95time (ms) | Pearson Correlation | 0.407 | 0.006 | 0.137 | 0.089 |
|  | Sig. (2-tailed) | <0.001 | 0.948 | 0.170 | 0.369 |
|  | N | 108 | 104 | 102 | 105 |
| b-wave 1.114time (ms) | Pearson Correlation | 0.405 | 0.129 | 0.199 | 0.180 |
|  | Sig. (2-tailed) | <0.001 | 0.188 | 0.044 | 0.065 |
|  | N | 109 | 105 | 103 | 106 |
| b-wave 1.204time (ms) | Pearson Correlation | 0.429 | 0.125 | 0.179 | 0.168 |
|  | Sig. (2-tailed) | <0.001 | 0.204 | 0.070 | 0.086 |
|  | N | 110 | 105 | 103 | 106 |
| b-wave -0.367amp (*u*V) | Pearson Correlation | -0.237 | 0.097 | 0.017 | 0.043 |
|  | Sig. (2-tailed) | 0.013 | 0.329 | 0.863 | 0.666 |
|  | N | 109 | 104 | 102 | 105 |
| b-wave -0.119amp (*u*V) | Pearson Correlation | -0.119 | 0.151 | 0.005 | 0.070 |
|  | Sig. (2-tailed) | 0.215 | 0.123 | 0.961 | 0.476 |
|  | N | 111 | 105 | 103 | 106 |
| b-wave 0.114amp (*u*V) | Pearson Correlation | -0.159 | 0.144 | 0.054 | 0.093 |
|  | Sig. (2-tailed) | 0.1 | 0.144 | 0.587 | 0.348 |
|  | N | 108 | 104 | 102 | 105 |
| b-wave 0.4amp (*u*V) | Pearson Correlation | -0.127 | 0.167 | 0.054 | 0.095 |
|  | Sig. (2-tailed) | 0.184 | 0.088 | 0.587 | 0.331 |
|  | N | 111 | 105 | 103 | 106 |
| b-wave 0.48amp (ISCEV) | Pearson Correlation | -0.129 | 0.170 | 0.054 | 0.097 |
|  | Sig. (2-tailed) | 0.183 | 0.083 | 0.590 | 0.323 |
|  | N | 109 | 105 | 103 | 106 |
| b-wave 0.6amp (*u*V) | Pearson Correlation | -0.179 | 0.111 | 0.025 | 0.058 |
|  | Sig. (2-tailed) | 0.063 | 0.260 | 0.802 | 0.558 |
|  | N | 109 | 104 | 102 | 105 |
| b-wave 0.8amp (*u*V) | Pearson Correlation | -0.05 | 0.089 | -0.006 | 0.033 |
|  | Sig. (2-tailed) | 0.605 | 0.365 | 0.951 | 0.739 |
|  | N | 110 | 105 | 103 | 106 |
| b-wave .95amp (*u*V) | Pearson Correlation | 0.012 | 0.042 | -0.068 | -0.029 |
|  | Sig. (2-tailed) | 0.899 | 0.673 | 0.497 | 0.769 |
|  | N | 108 | 104 | 102 | 105 |
| b-wave 1.114amp (*u*V) | Pearson Correlation | 0.081 | 0.118 | 0.030 | 0.065 |
|  | Sig. (2-tailed) | 0.404 | 0.232 | 0.763 | 0.509 |
|  | N | 109 | 105 | 103 | 106 |
| b-wave 1.204amp (*u*V) | Pearson Correlation | 0.024 | 0.042 | -0.067 | -0.033 |
|  | Sig. (2-tailed) | 0.800 | 0.670 | 0.498 | 0.734 |
|  | N | 110 | 105 | 103 | 106 |

**Photopic Negative Response parameters:**

| **ERG parameter** | | **Age (year)** | **BoW (%)** | **WoB (%)** | **Mean (%)** |
| --- | --- | --- | --- | --- | --- |
| p72_-0.367 (*u*V) | Pearson Correlation | -0.051 | -0.048 | -0.071 | -0.066 |
|  | Sig. (2-tailed) | 0.606 | 0.627 | 0.480 | 0.502 |
|  | N | 104 | 104 | 102 | 105 |
| p72_-0.119 (*u*V) | Pearson Correlation | 0.015 | -0.008 | -0.096 | -0.056 |
|  | Sig. (2-tailed) | 0.880 | 0.933 | 0.337 | 0.567 |
|  | N | 107 | 105 | 103 | 106 |
| p72_0.114 (*u*V) | Pearson Correlation | 0.128 | -0.207 | -0.253 | -0.25 |
|  | Sig. (2-tailed) | 0.192 | 0.035 | 0.010 | 0.010 |
|  | N | 105 | 104 | 102 | 105 |
| p72_0.4 (*u*V) | Pearson Correlation | -0.015 | -0.062 | -0.102 | -0.087 |
|  | Sig. (2-tailed) | 0.879 | 0.528 | 0.307 | 0.378 |
|  | N | 107 | 105 | 103 | 106 |
| p72_0.48 (ISCEV)  (*u*V) | Pearson Correlation | 0.088 | -0.002 | -0.117 | -0.069 |
|  | Sig. (2-tailed) | 0.374 | 0.981 | 0.241 | 0.487 |
|  | N | 105 | 104 | 102 | 105 |
| p72_0.6 (*u*V) | Pearson Correlation | 0.010 | -0.004 | -0.076 | -0.041 |
|  | Sig. (2-tailed) | 0.921 | 0.968 | 0.448 | 0.676 |
|  | N | 106 | 104 | 102 | 105 |
| p72_0.8 (*u*V) | Pearson Correlation | -0.005 | -0.029 | -0.11 | -0.075 |
|  | Sig. (2-tailed) | 0.961 | 0.772 | 0.269 | 0.445 |
|  | N | 107 | 105 | 103 | 106 |
| p72_0.95 (*u*V) | Pearson Correlation | -0.013 | -0.11 | -0.096 | -0.115 |
|  | Sig. (2-tailed) | 0.896 | 0.266 | 0.333 | 0.242 |
|  | N | 105 | 105 | 103 | 106 |
| p72_1.114 (*u*V) | Pearson Correlation | 0.081 | -0.104 | -0.160 | -0.141 |
|  | Sig. (2-tailed) | 0.41 | 0.293 | 0.105 | 0.148 |
|  | N | 106 | 105 | 103 | 106 |
| p72_1.204 (*u*V) | Pearson Correlation | 0.049 | -0.159 | -0.274 | -0.23 |
|  | Sig. (2-tailed) | 0.621 | 0.106 | 0.005 | 0.018 |
|  | N | 106 | 105 | 103 | 106 |
| PhNR_Tmin_-0.367 (*u*V) | Pearson Correlation | 0.018 | -0.074 | -0.051 | -0.069 |
|  | Sig. (2-tailed) | 0.857 | 0.456 | 0.607 | 0.483 |
|  | N | 104 | 104 | 102 | 105 |
| PhNR_Tmin_-0.119 (*u*V) | Pearson Correlation | 0.086 | -0.002 | -0.043 | -0.025 |
|  | Sig. (2-tailed) | 0.379 | 0.985 | 0.664 | 0.797 |
|  | N | 107 | 105 | 103 | 106 |
| PhNR_Tmin_0.114 (*u*V) | Pearson Correlation | -0.165 | -0.211 | -0.222 | -0.236 |
|  | Sig. (2-tailed) | 0.092 | 0.032 | 0.025 | 0.015 |
|  | N | 105 | 104 | 102 | 105 |
| PhNR_Tmin_0.4 (*u*V) | Pearson Correlation | 0.024 | -0.097 | -0.111 | -0.111 |
|  | Sig. (2-tailed) | 0.806 | 0.325 | 0.263 | 0.258 |
|  | N | 107 | 105 | 103 | 106 |
| PhNR_Tmin_0.48  (ISCEV) (*u*V) | Pearson Correlation | 0.071 | 0.055 | -0.037 | 0.007 |
|  | Sig. (2-tailed) | 0.473 | 0.576 | 0.715 | 0.947 |
|  | N | 105 | 104 | 102 | 105 |
| PhNR_Tmin_0.6 (*u*V) | Pearson Correlation | 0.024 | 0.050 | 0.080 | 0.070 |
|  | Sig. (2-tailed) | 0.806 | 0.613 | 0.424 | 0.477 |
|  | N | 107 | 104 | 102 | 105 |
| PhNR_Tmin_0.8 (*u*V) | Pearson Correlation | -0.052 | -0.054 | -0.085 | -0.077 |
|  | Sig. (2-tailed) | 0.598 | 0.585 | 0.394 | 0.435 |
|  | N | 107 | 105 | 103 | 106 |
| PhNR_Tmin_0.95 (*u*V) | Pearson Correlation | -0.069 | -0.106 | -0.092 | -0.107 |
|  | Sig. (2-tailed) | 0.483 | 0.281 | 0.357 | 0.276 |
|  | N | 105 | 105 | 103 | 106 |
| PhNR_Tmin_1.114 (*u*V) | Pearson Correlation | 0.125 | 0.002 | -0.028 | -0.016 |
|  | Sig. (2-tailed) | 0.201 | 0.984 | 0.781 | 0.871 |
|  | N | 106 | 105 | 103 | 106 |
| PhNR_Tmin_1.204 (*u*V) | Pearson Correlation | -0.064 | -0.181 | -0.244 | -0.226 |
|  | Sig. (2-tailed) | 0.517 | 0.064 | 0.013 | 0.020 |
|  | N | 106 | 105 | 103 | 106 |
| Tmin_-0.367 (ms) | Pearson Correlation | -0.052 | 0.011 | 0.036 | 0.020 |
|  | Sig. (2-tailed) | 0.603 | 0.912 | 0.722 | 0.840 |
|  | N | 104 | 104 | 102 | 105 |
| Tmin_-0.119 (ms) | Pearson Correlation | -0.010 | 0.164 | 0.199 | 0.183 |
|  | Sig. (2-tailed) | 0.917 | 0.095 | 0.044 | 0.061 |
|  | N | 107 | 105 | 103 | 106 |
| Tmin_0.114 (ms) | Pearson Correlation | -0.113 | -0.001 | 0.015 | 0.015 |
|  | Sig. (2-tailed) | 0.251 | 0.992 | 0.879 | 0.880 |
|  | N | 105 | 104 | 102 | 105 |
| Tmin_0.4 (ms) | Pearson Correlation | 0.010 | -0.130 | -0.098 | -0.110 |
|  | Sig. (2-tailed) | 0.921 | 0.186 | 0.323 | 0.260 |
|  | N | 107 | 105 | 103 | 106 |
| Tmin_0.48 (ISCEV) (ms) | Pearson Correlation | 0.117 | -0.112 | -0.006 | -0.057 |
|  | Sig. (2-tailed) | 0.233 | 0.259 | 0.951 | 0.564 |
|  | N | 105 | 104 | 102 | 105 |
| Tmin_0.6 (ms) | Pearson Correlation | 0.152 | 0.039 | 0.095 | 0.077 |
|  | Sig. (2-tailed) | 0.120 | 0.696 | 0.343 | 0.438 |
|  | N | 106 | 104 | 102 | 105 |
| Tmin_0.8 (ms) | Pearson Correlation | 0.074 | -0.017 | 0.051 | 0.025 |
|  | Sig. (2-tailed) | 0.450 | 0.865 | 0.612 | 0.797 |
|  | N | 107 | 105 | 103 | 106 |
| Tmin_0.95 (ms) | Pearson Correlation | 0.088 | -0.006 | 0.057 | 0.035 |
|  | Sig. (2-tailed) | 0.371 | 0.951 | 0.569 | 0.722 |
|  | N | 105 | 105 | 103 | 106 |
| Tmin_1.114 (ms) | Pearson Correlation | -0.056 | 0.067 | -0.006 | 0.037 |
|  | Sig. (2-tailed) | 0.571 | 0.498 | 0.955 | 0.709 |
|  | N | 106 | 105 | 103 | 106 |
| Tmin_1.204 (ms) | Pearson Correlation | 0.22 | -0.014 | -0.014 | -0.007 |
|  | Sig. (2-tailed) | 0.024 | 0.886 | 0.886 | 0.942 |
|  | N | 106 | 105 | 103 | 106 |

**Supplementary Table S5.** Comparisons of the ERG parameters between normal and abnormal motion coherence thresholds at each light strength.

The numbers in the left column presents the light strength of the flash in log cd.s.m^-2^; time=time-to-peak (ms); amp=amplitude (*u*V); ISCEV= International Society for Clinical Electrophysiology of Vision; p72 =PhNR amplitude at t=72ms (*u*V); Tmin= the time PhNR at minimum amplitude (ms); PhNR_Tmin= PhNR amplitude at Tmin (*u*V); N= number of subjects.

| **ERG Parameter at light strength (log cd.s.m^-2^)** | **Motion Coherence threshold (%)** | **N** | **Mean** | **Standard Deviation** | **Standard Error Mean** | **t** | **p-value** |
| --- | --- | --- | --- | --- | --- | --- | --- |
| a-wave -0.367time (ms) | Normal | 126 | 14.07 | 2.03 | 0.18 |  |  |
|  | Abnormal | 67 | 13.52 | 2.32 | 0.28 | 1.649 | 0.102 |
| a-wave -0.119time (ms) | Normal | 126 | 13.4 | 1.68 | 0.15 |  |  |
|  | Abnormal | 69 | 12.74 | 1.71 | 0.21 | 2.576 | 0.011 |
| a-wave 0.114time (ms) | Normal | 125 | 12.9 | 1.13 | 0.1 |  |  |
|  | Abnormal | 65 | 12.66 | 1.23 | 0.15 | 1.325 | 0.188 |
| a-wave 0.4time (ms) | Normal | 126 | 12.28 | 1.13 | 0.1 |  |  |
|  | Abnormal | 68 | 11.85 | 1.14 | 0.14 | 2.489 | 0.014 |
| a-wave 0.48time (ISCEV) (ms) | Normal | 125 | 11.71 | 1.38 | 0.12 |  |  |
|  | Abnormal | 67 | 11.43 | 1.23 | 0.15 | 1.447 | 0.15 |
| a-wave 0.6time (ms) | Normal | 126 | 11.9 | 1.09 | 0.1 |  |  |
|  | Abnormal | 68 | 11.58 | 1.08 | 0.13 | 1.961 | 0.052 |
| a-wave 0.8time (ms) | Normal | 126 | 11.51 | 0.95 | 0.08 |  |  |
|  | Abnormal | 68 | 11.16 | 1.54 | 0.19 | 1.666 | 0.099 |
| a-wave 0.95time (ms) | Normal | 125 | 11.56 | 1.17 | 0.1 |  |  |
|  | Abnormal | 67 | 11.36 | 1.38 | 0.17 | 1.01 | 0.315 |
| a-wave 1.114time (ms) | Normal | 126 | 11.4 | 1.08 | 0.1 |  |  |
|  | Abnormal | 66 | 11.17 | 0.9 | 0.11 | 1.503 | 0.135 |
| a-wave 1.204time (ms) | Normal | 126 | 11.55 | 1.01 | 0.09 |  |  |
|  | Abnormal | 66 | 11.16 | 1.07 | 0.13 | 2.436 | 0.016 |
| a-wave -0.367amp (*u*V) | Normal | 126 | 4.42 | 1.92 | 0.17 |  |  |
|  | Abnormal | 67 | 3.92 | 1.86 | 0.23 | 1.76 | 0.081 |
| a-wave -0.119amp (*u*V) | Normal | 126 | 5.09 | 2.06 | 0.18 |  |  |
|  | Abnormal | 69 | 4.69 | 1.97 | 0.24 | 1.342 | 0.182 |
| a-wave 0.114amp (*u*V) | Normal | 125 | 5.63 | 2.01 | 0.18 |  |  |
|  | Abnormal | 65 | 5.88 | 2.8 | 0.35 | -0.663 | 0.509 |
| a-wave 0.4amp (*u*V) | Normal | 126 | 6.71 | 2.13 | 0.19 |  |  |
|  | Abnormal | 68 | 6.25 | 2.12 | 0.26 | 1.456 | 0.148 |
| a-wave 0.48amp (ISCEV) (*u*V) | Normal | 125 | 7.78 | 2.65 | 0.24 |  |  |
|  | Abnormal | 67 | 7.95 | 3.09 | 0.38 | -0.381 | 0.704 |
| a-wave 0.6amp (*u*V) | Normal | 126 | 7.67 | 2.33 | 0.21 |  |  |
|  | Abnormal | 68 | 7.39 | 2.51 | 0.3 | 0.764 | 0.446 |
| a-wave 0.8amp (*u*V) | Normal | 126 | 8.3 | 2.72 | 0.24 |  |  |
|  | Abnormal | 68 | 8.08 | 2.71 | 0.33 | 0.534 | 0.594 |
| a-wave 0.95amp (*u*V) | Normal | 125 | 9.12 | 3.06 | 0.27 |  |  |
|  | Abnormal | 67 | 8.17 | 3.35 | 0.41 | 1.921 | 0.057 |
| a-wave 1.114amp (*u*V) | Normal | 126 | 10.02 | 3.03 | 0.27 |  |  |
|  | Abnormal | 66 | 9.01 | 3.34 | 0.41 | 2.058 | 0.042 |
| a-wave 1.204amp (*u*V) | Normal | 126 | 10.49 | 3.41 | 0.3 |  |  |
|  | Abnormal | 66 | 9.3 | 3.39 | 0.42 | 2.314 | 0.022 |
| b-wave -0.367time (ms) | Normal | 126 | 22.42 | 2.54 | 0.23 |  |  |
|  | Abnormal | 66 | 22.94 | 2.26 | 0.28 | -1.458 | 0.147 |
| b-wave -0.119time (ms) | Normal | 126 | 23.7 | 1.42 | 0.13 |  |  |
|  | Abnormal | 69 | 23.97 | 1.68 | 0.2 | -1.142 | 0.256 |
| b-wave 0.114time (ms) | Normal | 125 | 24.82 | 0.97 | 0.09 |  |  |
|  | Abnormal | 67 | 25.37 | 2 | 0.24 | -2.11 | 0.038 |
| b-wave 0.4time (ms) | Normal | 126 | 26.14 | 0.98 | 0.09 |  |  |
|  | Abnormal | 70 | 26.59 | 1.27 | 0.15 | -2.56 | 0.012 |
| b-wave 0.48time (ISCEV) (ms) | Normal | 125 | 28.57 | 1.36 | 0.12 |  |  |
|  | Abnormal | 67 | 28.52 | 1.55 | 0.19 | 0.234 | 0.815 |
| b-wave 0.6time (ms) | Normal | 126 | 27.51 | 1.21 | 0.11 |  |  |
|  | Abnormal | 67 | 27.95 | 1.53 | 0.19 | -2.008 | 0.047 |
| b-wave 0.8time (ms) | Normal | 126 | 29 | 1.3 | 0.12 |  |  |
|  | Abnormal | 68 | 29.13 | 1.55 | 0.19 | -0.578 | 0.564 |
| b-wave 0.95time (ms) | Normal | 125 | 29.66 | 2.47 | 0.22 |  |  |
|  | Abnormal | 67 | 29.81 | 1.48 | 0.18 | -0.537 | 0.592 |
| b-wave 1.114time (ms) | Normal | 126 | 30.64 | 1.66 | 0.15 |  |  |
|  | Abnormal | 66 | 30.78 | 1.72 | 0.21 | -0.548 | 0.585 |
| b-wave 1.204time (ms) | Normal | 126 | 31.07 | 1.77 | 0.16 |  |  |
|  | Abnormal | 66 | 31.17 | 2.04 | 0.25 | -0.348 | 0.729 |
| b-wave -0.367amp (*u*V) | Normal | 126 | 13.43 | 4.65 | 0.41 |  |  |
|  | Abnormal | 66 | 13.21 | 4.32 | 0.53 | 0.323 | 0.747 |
| b-wave -0.119amp (*u*V) | Normal | 126 | 17.61 | 5.08 | 0.45 |  |  |
|  | Abnormal | 69 | 18.06 | 4.67 | 0.56 | -0.627 | 0.532 |
| b-wave 0.114amp (*u*V) | Normal | 125 | 27.17 | 7.27 | 0.65 |  |  |
|  | Abnormal | 67 | 28.78 | 8.41 | 1.03 | -1.325 | 0.188 |
| b-wave 0.4amp (*u*V) | Normal | 126 | 34.52 | 9.34 | 0.83 |  |  |
|  | Abnormal | 70 | 33.82 | 10.11 | 1.21 | 0.472 | 0.638 |
| b-wave 0.48amp (ISCEV) (*u*V) | Normal | 125 | 32.41 | 9.84 | 0.88 |  |  |
|  | Abnormal | 67 | 34.19 | 10.07 | 1.23 | -1.181 | 0.24 |
| b-wave 0.6amp (*u*V) | Normal | 126 | 35.03 | 9.72 | 0.87 |  |  |
|  | Abnormal | 67 | 34.76 | 10.57 | 1.29 | 0.174 | 0.862 |
| b-wave 0.8amp (*u*V) | Normal | 126 | 32.62 | 9.02 | 0.8 |  |  |
|  | Abnormal | 68 | 33.57 | 9.51 | 1.15 | -0.682 | 0.496 |
| b-wave 0.95amp (*u*V) | Normal | 125 | 31.34 | 8.63 | 0.77 |  |  |
|  | Abnormal | 67 | 31.85 | 9.68 | 1.18 | -0.358 | 0.721 |
| b-wave 1.114amp (*u*V) | Normal | 126 | 31.1 | 8.55 | 0.76 |  |  |
|  | Abnormal | 66 | 32.27 | 9.34 | 1.15 | -0.852 | 0.396 |
| b-wave 1.204amp (*u*V) | Normal | 126 | 29.9 | 9 | 0.8 |  |  |
|  | Abnormal | 66 | 29.6 | 8.72 | 1.07 | 0.227 | 0.821 |
| Tmin_-0.367 (ms) | Normal | 126 | 75.15 | 15.97 | 1.42 |  |  |
|  | Abnormal | 66 | 77 | 17.12 | 2.11 | -0.726 | 0.235 |
| Tmin_-0.119 (ms) | Normal | 126 | 71.81 | 15.44 | 1.38 |  |  |
|  | Abnormal | 69 | 76.49 | 16.26 | 1.96 | -1.956 | 0.026 |
| Tmin_0.114 (ms) | Normal | 125 | 72.52 | 15.5 | 1.39 |  |  |
|  | Abnormal | 68 | 75.88 | 16.85 | 2.04 | -1.359 | 0.088 |
| Tmin_0.4 (ms) | Normal | 126 | 73.91 | 14.61 | 1.3 |  |  |
|  | Abnormal | 70 | 73.74 | 14.95 | 1.79 | 0.078 | 0.469 |
| Tmin_0.48 (ISCEV) (ms) | Normal | 126 | 73.15 | 12.6 | 1.12 |  |  |
|  | Abnormal | 67 | 73.21 | 13.15 | 1.61 | -0.028 | 0.489 |
| Tmin_0.6 (ms) | Normal | 126 | 71.49 | 12.94 | 1.15 |  |  |
|  | Abnormal | 68 | 75.43 | 14.35 | 1.74 | -1.888 | 0.031 |
| Tmin_0.8 (ms) | Normal | 126 | 74.36 | 14.35 | 1.28 |  |  |
|  | Abnormal | 70 | 77.66 | 13.97 | 1.67 | -1.568 | 0.059 |
| Tmin_0.95 (ms) | Normal | 126 | 72.56 | 14.27 | 1.27 |  |  |
|  | Abnormal | 68 | 74.42 | 14.94 | 1.81 | -0.843 | 0.2 |
| Tmin_1.114 (ms) | Normal | 126 | 74.42 | 15.23 | 1.36 |  |  |
|  | Abnormal | 68 | 76.58 | 14.01 | 1.7 | -0.992 | 0.161 |
| Tmin_1.204 (ms) | Normal | 126 | 74.62 | 14.43 | 1.29 |  |  |
|  | Abnormal | 67 | 75.13 | 15.84 | 1.94 | -0.218 | 0.414 |
| p72_-0.367 (*u*V) | Normal | 126 | 3.14 | 3.42 | 0.3 |  |  |
|  | Abnormal | 66 | 2.52 | 3.35 | 0.41 | 1.222 | 0.112 |
| p72_-0.119 (*u*V) | Normal | 126 | 4.19 | 3.79 | 0.34 |  |  |
|  | Abnormal | 69 | 4.57 | 3.74 | 0.45 | -0.677 | 0.25 |
| p72_0.114 (*u*V) | Normal | 125 | 4.85 | 3.69 | 0.33 |  |  |
|  | Abnormal | 68 | 3.92 | 3.75 | 0.45 | 1.655 | 0.049 |
| p72_0.4 (*u*V) | Normal | 126 | 5.57 | 3.25 | 0.29 |  |  |
|  | Abnormal | 70 | 5.38 | 3.67 | 0.44 | 0.359 | 0.36 |
| p72_0.48 (ISCEV) (*u*V) | Normal | 126 | 6.29 | 3.95 | 0.35 |  |  |
|  | Abnormal | 67 | 6.24 | 4.57 | 0.56 | 0.72 | 0.472 |
| p72_0.6 (*u*V) | Normal | 126 | 6.22 | 3.58 | 0.32 |  |  |
|  | Abnormal | 68 | 5.88 | 3.53 | 0.43 | 0.647 | 0.259 |
| p72_0.8 (*u*V) | Normal | 126 | 6.99 | 4.3 | 0.38 |  |  |
|  | Abnormal | 70 | 7.23 | 4.13 | 0.49 | -0.385 | 0.35 |
| p72_0.95 (*u*V) | Normal | 126 | 7.58 | 4.6 | 0.41 |  |  |
|  | Abnormal | 68 | 7.19 | 5.09 | 0.62 | 0.523 | 0.301 |
| p72_1.114 (*u*V) | Normal | 126 | 7.99 | 4.25 | 0.38 |  |  |
|  | Abnormal | 68 | 7.03 | 4.4 | 0.53 | 1.467 | 0.072 |
| p72_1.204 (*u*V) | Normal | 126 | 8.66 | 5.1 | 0.45 |  |  |
|  | Abnormal | 67 | 7.09 | 4.21 | 0.51 | 2.293 | 0.012 |
| PhNR_Tmin_-0.367 (*u*V) | Normal | 126 | 5.58 | 3.7 | 0.33 |  |  |
|  | Abnormal | 66 | 5.15 | 3.43 | 0.42 | 0.792 | 0.215 |
| PhNR_Tmin_-0.119 (*u*V) | Normal | 126 | 6.62 | 3.81 | 0.34 |  |  |
|  | Abnormal | 69 | 6.97 | 4.32 | 0.52 | -0.559 | 0.288 |
| PhNR_Tmin_0.114 (*u*V) | Normal | 125 | 7.03 | 3.46 | 0.31 |  |  |
|  | Abnormal | 68 | 6.7 | 4.33 | 0.52 | 0.529 | 0.299 |
| PhNR_Tmin_0.4 (*u*V) | Normal | 126 | 7.75 | 3.69 | 0.33 |  |  |
|  | Abnormal | 70 | 7.68 | 3.77 | 0.45 | 0.124 | 0.451 |
| PhNR_Tmin_0.48 (ISCEV) | Normal | 126 | 8.26 | 4.12 | 0.37 |  |  |
| (*u*V) | Abnormal | 67 | 8.61 | 4.44 | 0.54 | -0.547 | 0.293 |
| PhNR_Tmin_0.6 (*u*V) | Normal | 126 | 8.04 | 3.81 | 0.34 |  |  |
|  | Abnormal | 68 | 8.48 | 4.33 | 0.53 | -0.702 | 0.242 |
| PhNR_Tmin_0.8 (*u*V) | Normal | 126 | 9.3 | 4.81 | 0.43 |  |  |
|  | Abnormal | 70 | 9.95 | 3.97 | 0.47 | -1.019 | 0.155 |
| PhNR_Tmin_0.95 (*u*V) | Normal | 126 | 9.74 | 5.49 | 0.49 |  |  |
|  | Abnormal | 68 | 9.46 | 5.53 | 0.67 | 0.341 | 0.367 |
| PhNR_Tmin_1.114 (*u*V) | Normal | 126 | 9.61 | 8.1 | 0.72 |  |  |
|  | Abnormal | 68 | 9.41 | 4.23 | 0.51 | 0.228 | 0.41 |
| PhNR_Tmin_1.204 (*u*V) | Normal | 126 | 11.12 | 6.14 | 0.55 |  |  |
|  | Abnormal | 67 | 9.76 | 4.48 | 0.55 | 1.758 | 0.04 |

# **Supplementary Table S6.** Correlations of full-scale IQ, ADOS and autism severity scores with ERG parameters in ASD families.

N=Number of cases; FSIQ=Full-scale intellectual quotient; ADOS=Autism Diagnostic Observation Schedule total score; Autism Severity=Autism severity score. The numbers in the left column presents the light strength of the flash in log cd.s.m^-2^; time (ms)=time (ms)-to-peak (ms); amp=amplitude (*u*V); ISCEV= International Society for Clinical Electrophysiology of Vision; p72 =PhNR amplitude at t=72ms (*u*V); Tmin= the time (ms) PhNR at minimum amplitude (ms); PhNR_Tmin= PhNR amplitude at Tmin (*u*V). Only those with a p-value <0.05 are presented below.

| **ERG Parameter** |  | **FSIQ** | **ADOS** | **Autism Severity** |
| --- | --- | --- | --- | --- |
| a-wave 0.4time (ms) | Pearson Correlation (N) | 0.347 (39) | 0.013 (39) | 0.017 |
|  | p-value | 0.03 | 0.936 | 0.916 |
| b-wave 0.48time (ms)) | Pearson Correlation (N) | 0.378 (39) | -0.084 (38) | -0.073 (40) |
| (ISCEV | p-value | 0.018 | 0.617 | 0.652 |
| b-wave 0.8time (ms) | Pearson Correlation (N) | 0.359 (39) | -0.059 (39) | 0.474 (41) |
|  | p-value | 0.025 | 0.329 | 0.613 |
| b-wave 1.204time (ms) | Pearson Correlation (N) | 0.343 (39) | -0.156 (39) | -0.105 (41) |
|  | p-value | 0.032 | 0.344 | 0.514 |
| b-wave -0.367amp (*u*V) | Pearson Correlation (N) | 0.129 (39) | 0.346 (38) | 0.264 (40) |
|  | p-value | 0.435 | 0.033 | 0.099 |
| p72_0.48 (ISCEV) (uV) | Pearson Correlation (N) | 0.138 (38) | -0.376 (37) | -0.374 (39) |
|  | p-value | 0.409 | 0.022 | 0.019 |
| PhNR_Tmin_0.48 (ISCEV) | Pearson Correlation (N) | 0.223 (38) | -0.322 (37) | -0.354 (39) |
| (*u*V) | p-value | 0.178 | 0.052 | 0.027 |
| PhNR_Tmin_1.204 (uV) | Pearson Correlation (N) | 0.093 (38) | -0.440 (38) | -0.412 (40) |
|  | p-value | 0.58 | 0.006 | 0.008 |

**Supplementary Table S7.** Bayesian analysis to compare the ERG parameters between normal and abnormal motion coherence thresholds at each phenotypic variable and light strength.

The numbers in the left column presents phenotypic variables and the light strength of the flash in log cd.s.m^-2^; Age= age of participant in year; FSIQ= full-scale Intellectual quotient; ADOS= Autism Diagnostic Observation Schedule total score; Autism Severity=Autism severity score; time (ms)=time (ms)-to-peak (ms); amp=amplitude (*u*V); ISCEV= International Society for Clinical Electrophysiology of Vision; p72 =PhNR amplitude at t=72ms (*u*V); Tmin= the time (ms) PhNR at minimum amplitude (ms); PhNR_Tmin= PhNR amplitude at Tmin (*u*V); N= number of subjects. SD= Standard deviation; SEM=Standard Error Mean. BF_10_=Bayes Factor (BF_10_ between 3 to 10 indicates moderate evidence

for an effect.

| **Phenotypic variable** | **% Motion Coherence threshold** | **N** | **Mean** | **SD** | **SEM** | **BF_10_** | **t** | **p-value** |
| --- | --- | --- | --- | --- | --- | --- | --- | --- |
| Age (year) | Normal | 225 | 23.90 | 15.73 | 1.03 |  |  |  |
|  | Abnormal | 44 | 24.06 | 15.61 | 2.23 | 8.15 | 0.065 | 0.949 |
| FSIQ | Normal | 19 | 105.85 | 18.17 | 4.17 |  |  |  |
|  | Abnormal | 19 | 91.21 | 17.549 | 4.026 | 0.31 | -2.525 | 0.016 |
| ADOS | Normal | 19 | 10.53 | 4.86 | 1.11 |  |  |  |
|  | Abnormal | 21 | 12.71 | 4.56 | 0.996 | 1.70 | 1.469 | 0.150 |
| Autism Severity | Normal | 19 | 6.32 | 1.89 | 0.43 |  |  |  |
|  | Abnormal | 21 | 7.10 | 1.81 | 0.40 | 1.996 | 1.331 | 0.191 |
| Iris Index Colour | Normal | 146 | 1.30 | 0.13 | 0.01 |  |  |  |
|  | Abnormal | 72 | 1.26 | 0.11 | 0.01 | 0.653 | -2.337 | 0.02 |
| **ERG Parameter at light strength (log cd.s.m^-2^)** | |  |  |  |  |  |  |  |
| a-wave -0.367time (ms) | Normal | 147 | 14.08 | 2.03 | 0.17 |  |  |  |
|  | Abnormal | 67 | 13.52 | 2.32 | 0.28 | 1.817 | -1.806 | 0.072 |
| a-wave -0.119time (ms) | Normal | 148 | 13.52 | 1.66 | 0.14 |  |  |  |
|  | Abnormal | 69 | 12.74 | 1.71 | 0.21 | 0.076 | -3.166 | 0.002 |
| a-wave 0.114time (ms) | Normal | 147 | 12.87 | 1.16 | 0.10 |  |  |  |
|  | Abnormal | 65 | 12.66 | 1.23 | 0.15 | 4.300 | -1.200 | 0.231 |
| a-wave 0.4time (ms) | Normal | 148 | 12.33 | 1.12 | 0.09 |  |  |  |
|  | Abnormal | 68 | 11.85 | 1.14 | 0.14 | 0.148 | -2.931 | 0.004 |
| a-wave 0.48time (ms) (ISCEV) | Normal | 146 | 11.74 | 1.35 | 0.11 |  |  |  |
|  | Abnormal | 67 | 11.43 | 1.23 | 0.15 | 2.566 | -1.593 | 0.063 |
| a-wave 0.6time (ms) | Normal | 148 | 11.88 | 1.10 | 0.09 |  |  |  |
|  | Abnormal | 68 | 11.58 | 1.08 | 0.13 | 1.641 | -1.867 | 0.030 |
| a-wave 0.8time (ms) | Normal | 148 | 11.54 | 0.94 | 0.08 |  |  |  |
|  | Abnormal | 68 | 11.16 | 1.54 | 0.19 | 0.898 | -2.181 | 0.161 |
| a-wave 0.95time (ms) | Normal | 147 | 11.61 | 1.15 | 0.10 |  |  |  |
|  | Abnormal | 67 | 11.36 | 1.38 | 0.17 | 3.354 | -1.407 | 0.058 |
| a-wave 1.114time (ms) | Normal | 148 | 11.46 | 1.08 | 0.09 |  |  |  |
|  | Abnormal | 66 | 11.17 | 0.9 | 0.11 | 1.524 | -1.904 | 0.135 |
| a-wave 1.204time (ms) | Normal | 148 | 11.67 | 1.17 | 0.10 |  |  |  |
|  | Abnormal | 66 | 11.16 | 1.07 | 0.13 | 0.119 | -3.009 | 0.003 |
| a-wave -0.367amp (*u*V) | Normal | 147 | 4.31 | 1.94 | 0.16 |  |  |  |
|  | Abnormal | 67 | 3.92 | 1.86 | 0.23 | 3.536 | -1.367 | 0.173 |
| a-wave -0.119amp (*u*V) | Normal | 148 | 4.95 | 2.05 | 0.17 |  |  |  |
|  | Abnormal | 69 | 4.69 | 1.97 | 0.24 | 5.994 | -0.889 | 0.375 |
| a-wave 0.114amp (*u*V) | Normal | 147 | 5.49 | 1.98 | 0.16 |  |  |  |
|  | Abnormal | 65 | 5.88 | 2.8 | 0.35 | 4.371 | 1.186 | 0.237 |
| a-wave 0.4amp (*u*V) | Normal | 148 | 6.53 | 2.11 | 0.17 |  |  |  |
|  | Abnormal | 68 | 6.25 | 2.12 | 0.26 | 5.830 | -0.916 | 0.361 |
| a-wave 0.48amp (ISCEV) (*u*V) | Normal | 146 | 7.59 | 2.59 | 0.22 |  |  |  |
|  | Abnormal | 67 | 7.95 | 3.09 | 0.38 | 5.896 | 0.895 | 0.372 |
| a-wave 0.6amp (*u*V) | Normal | 148 | 7.48 | 2.35 | 0.19 |  |  |  |
|  | Abnormal | 68 | 7.39 | 2.51 | 0.3 | 8.503 | -0.234 | 0.815 |
| a-wave 0.8amp (*u*V) | Normal | 148 | 8.03 | 2.69 | 0.22 |  |  |  |
|  | Abnormal | 68 | 8.08 | 2.71 | 0.33 | 8.662 | 0.129 | 0.898 |
| a-wave 0.95amp (*u*V) | Normal | 147 | 8.90 | 3.13 | 0.26 |  |  |  |
|  | Abnormal | 67 | 8.17 | 3.35 | 0.41 | 2.811 | -1.532 | 0.127 |
| a-wave 1.114amp (*u*V) | Normal | 148 | 10.02 | 3.03 | 0.27 |  |  |  |
|  | Abnormal | 66 | 9.01 | 3.34 | 0.41 | 2.491 | -1.610 | 0.109 |
| a-wave 1.204amp (*u*V) | Normal | 148 | 10.24 | 3.38 | 0.28 |  |  |  |
|  | Abnormal | 66 | 9.3 | 3.39 | 0.42 | 1.595 | -1.879 | 0.062 |
| b-wave -0.367time (ms) | Normal | 148 | 22.43 | 2.39 | 0.20 |  |  |  |
|  | Abnormal | 66 | 22.94 | 2.26 | 0.28 | 3.037 | 1.476 | 0.141 |
| b-wave -0.119time (ms) | Normal | 148 | 23.67 | 1.45 | 0.13 |  |  |  |
|  | Abnormal | 69 | 23.97 | 1.68 | 0.12 | 3.672 | 1.346 | 0.180 |
| b-wave 0.114time (ms) | Normal | 147 | 24.81 | 0.96 | 0.08 |  |  |  |
|  | Abnormal | 67 | 25.37 | 2.00 | 0.24 | 0.223 | 2.776 | 0.006 |
| b-wave 0.4time (ms) | Normal | 148 | 26.01 | 0.94 | 0.08 |  |  |  |
|  | Abnormal | 70 | 26.59 | 1.27 | 0.15 | 0.064 | 3.229 | 0.001 |
| b-wave 0.48time (ms) (ISCEV) | Normal | 146 | 28.49 | 1.32 | 0.11 |  |  |  |
|  | Abnormal | 67 | 28.52 | 1.55 | 0.19 | 8.599 | 0.131 | 0.896 |
| b-wave 0.6time (ms) | Normal | 148 | 27.49 | 1.18 | 0.10 |  |  |  |
|  | Abnormal | 67 | 27.95 | 1.53 | 0.19 | 0.569 | 2.391 | 0.018 |
| b-wave 0.8time (ms) | Normal | 148 | 28.94 | 1.26 | 0.10 |  |  |  |
|  | Abnormal | 68 | 29.13 | 1.55 | 0.19 | 5.616 | 0.957 | 0.340 |
| b-wave 0.95time (ms) | Normal | 147 | 29.55 | 2.38 | 0.20 |  |  |  |
|  | Abnormal | 67 | 29.81 | 1.48 | 0.18 | 6.103 | 0.855 | 0.394 |
| b-wave 1.114time (ms) | Normal | 148 | 30.57 | 1.58 | 0.13 |  |  |  |
|  | Abnormal | 66 | 30.78 | 1.72 | 0.21 | 5.898 | 0.891 | 0.374 |
| b-wave 1.204time (ms) | Normal | 148 | 31.03 | 1.68 | 0.14 |  |  |  |
|  | Abnormal | 66 | 31.17 | 2.04 | 0.25 | 7.653 | 0.503 | 0.616 |
| b-wave -0.367amp (*u*V) | Normal | 148 | 13.02 | 4.86 | 0.40 |  |  |  |
|  | Abnormal | 66 | 13.21 | 4.32 | 0.53 | 8.320 | 0.282 | 0.778 |
| b-wave -0.119amp (*u*V) | Normal | 148 | 17.11 | 5.04 | 0.44 |  |  |  |
|  | Abnormal | 69 | 18.06 | 4.67 | 0.56 | 4.094 | 1.258 | 0.210 |
| b-wave 0.114amp (*u*V) | Normal | 147 | 26.72 | 8.03 | 0.66 |  |  |  |
|  | Abnormal | 67 | 28.78 | 8.41 | 1.03 | 2.117 | 1.715 | 0.088 |
| b-wave 0.4amp (*u*V) | Normal | 148 | 34.00 | 9.91 | 0.82 |  |  |  |
|  | Abnormal | 70 | 33.82 | 10.11 | 1.21 | 8.754 | -0.120 | 0.905 |
| b-wave 0.48amp (ISCEV) (*u*V) | Normal | 146 | 32.01 | 9.84 | 0.81 |  |  |  |
|  | Abnormal | 67 | 34.19 | 10.07 | 1.23 | 2.962 | 1.496 | 0.136 |
| b-wave 0.6amp (*u*V) | Normal | 148 | 34.42 | 9.86 | 0.81 |  |  |  |
|  | Abnormal | 67 | 34.76 | 10.57 | 1.29 | 8.465 | 0.232 | 0.816 |
| b-wave 0.8amp (*u*V) | Normal | 148 | 32.15 | 9.04 | 0.74 |  |  |  |
|  | Abnormal | 68 | 33.57 | 9.51 | 1.15 | 5.075 | 1.061 | 0.290 |
| b-wave 0.95amp (*u*V) | Normal | 147 | 30.94 | 8.86 | 0.73 |  |  |  |
|  | Abnormal | 67 | 31.85 | 9.68 | 1.18 | 6.970 | 0.675 | 0.501 |
| b-wave 1.114amp (*u*V) | Normal | 148 | 30.73 | 8.63 | 0.71 |  |  |  |
|  | Abnormal | 66 | 32.27 | 9.34 | 1.15 | 4.450 | 1.175 | 0.241 |
| b-wave 1.204amp (*u*V) | Normal | 148 | 29.56 | 9.20 | 0.76 |  |  |  |
|  | Abnormal | 66 | 29.6 | 8.72 | 1.07 | 8.643 | 0.026 | 0.979 |
| Tmin_-0.367 (ms) | Normal | 140 | 75.35 | 16.10 | 1.36 |  |  |  |
|  | Abnormal | 66 | 77 | 17.12 | 2.11 | 6.902 | 0.671 | 0.503 |
| Tmin_-0.119 (ms) | Normal | 140 | 71.50 | 15.17 | 1.28 |  |  |  |
|  | Abnormal | 69 | 76.49 | 16.26 | 1.96 | 0.898 | 2.180 | 0.030 |
| Tmin_0.114 (ms) | Normal | 139 | 72.08 | 15.33 | 1.30 |  |  |  |
|  | Abnormal | 68 | 75.88 | 16.85 | 2.04 | 2.470 | 1.616 | 0.108 |
| Tmin_0.4 (ms) | Normal | 139 | 73.21 | 12.43 | 1.05 |  |  |  |
|  | Abnormal | 70 | 73.74 | 14.95 | 1.79 | 8.650 | 0.145 | 0.885 |
| Tmin_0.48 (ISCEV) | Normal | 139 | 73.21 | 12.43 | 1.05 |  |  |  |
| (ms) | Abnormal | 67 | 73.21 | 13.15 | 1.61 | 8.606 | 0.001 | 0.990 |
| Tmin_0.6 (ms) | Normal | 140 | 71.48 | 12.45 | 1.05 |  |  |  |
|  | Abnormal | 68 | 75.43 | 14.35 | 1.74 | 1.181 | 2.041 | 0.043 |
| Tmin_0.8 (ms) | Normal | 140 | 74.25 | 14.21 | 1.20 |  |  |  |
|  | Abnormal | 70 | 77.66 | 13.97 | 1.67 | 2.377 | 1.647 | 0.101 |
| Tmin_0.95 (ms) | Normal | 140 | 72.94 | 13.98 | 1.18 |  |  |  |
|  | Abnormal | 68 | 74.42 | 14.94 | 1.81 | 6.829 | 0.701 | 0.484 |
| Tmin_1.114 (ms) | Normal | 140 | 75.13 | 15.40 | 1.30 |  |  |  |
|  | Abnormal | 68 | 76.58 | 14.01 | 1.7 | 7.048 | 0.653 | 0.514 |
| Tmin_1.204 (ms) | Normal | 140 | 74.57 | 14.05 | 1.19 |  |  |  |
|  | Abnormal | 67 | 75.13 | 15.84 | 1.94 | 8.351 | 0.254 | 0.800 |
| p72_-0.367 (*u*V) | Normal | 140 | -3.08 | 3.29 | 0.28 |  |  |  |
|  | Abnormal | 66 | -2.52 | 3.35 | 0.41 | 4.566 | 1.145 | 0.254 |
| p72_-0.119 (*u*V) | Normal | 140 | -4.07 | 3.67 | 0.31 |  |  |  |
|  | Abnormal | 69 | -4.57 | 3.74 | 0.45 | 5.728 | -0.931 | 0.353 |
| p72_0.114 (*u*V) | Normal | 139 | -4.75 | 3.62 | 0.31 |  |  |  |
|  | Abnormal | 68 | -3.92 | 3.75 | 0.46 | 2.801 | 1.532 | 0.127 |
| p72_0.4 (*u*V) | Normal | 140 | -5.45 | 3.18 | 0.27 |  |  |  |
|  | Abnormal | 70 | -5.38 | 3.67 | 0.44 | 8.637 | 0.155 | 0.877 |
| p72_0.48 (ISCEV) | Normal | 139 | -6.16 | 3.80 | 0.32 |  |  |  |
| (*u*V) | Abnormal | 67 | -6.24 | 4.57 | 0.56 | 8.529 | -0.137 | 0.891 |
| p72_0.6 (*u*V) | Normal | 140 | -6.23 | 3.54 | 0.30 |  |  |  |
|  | Abnormal | 68 | -5.88 | 3.53 | 0.43 | 6.964 | 0.672 | 0.502 |
| p72_0.8 (*u*V) | Normal | 140 | -6.98 | 4.82 | 0.41 |  |  |  |
|  | Abnormal | 70 | -7.23 | 4.13 | 0.49 | 8.168 | -0.374 | 0.709 |
| p72_0.95 (*u*V) | Normal | 140 | -7.52 | 4.75 | 0.40 |  |  |  |
|  | Abnormal | 68 | -7.19 | 5.09 | 0.62 | 7.809 | 0.462 | 0.644 |
| p72_1.114 (*u*V) | Normal | 140 | -7.86 | 4.21 | 0.36 |  |  |  |
|  | Abnormal | 68 | -7.03 | 4.40 | 0.53 | 3.802 | 1.308 | 0.192 |
| p72_1.204 (*u*V) | Normal | 140 | -8.66 | 5.10 | 0.443 |  |  |  |
|  | Abnormal | 67 | -7.09 | 3.79 | 0.463 | 2.200 | 2.003 | 0.049 |
| PhNR_Tmin_-0.367 | Normal | 140 | -5.44 | 3.56 | 0.30 |  |  |  |
| (*u*V) | Abnormal | 66 | -5.15 | 3.43 | 0.42 | 7.399 | 0.553 | 0.581 |
| PhNR_Tmin_-0.119 | Normal | 140 | -6.40 | 3.73 | 0.32 |  |  |  |
| (*u*V) | Abnormal | 69 | -6.97 | 4.32 | 0.52 | 5.448 | -0.986 | 0.326 |
| PhNR_Tmin_0.114 | Normal | 139 | -6.85 | 3.52 | 0.30 |  |  |  |
| (*u*V) | Abnormal | 68 | -6.70 | 4.33 | 0.52 | 8.371 | .259 | 0.796 |
| PhNR_Tmin_0.4 (*u*V) | Normal | 140 | -7.53 | 3.63 | 0.31 |  |  |  |
|  | Abnormal | 70 | -7.68 | 3.77 | 0.45 | 8.407 | -0.283 | 0.778 |
| PhNR_Tmin_0.48 | Normal | 139 | -8.04 | 4.01 | 0.34 |  |  |  |
| (ISCEV) (*u*V) | Abnormal | 67 | -8.61 | 4.44 | 0.54 | 5.651 | -0.935 | 0.351 |
| PhNR_Tmin_0.6 (*u*V) | Normal | 140 | -7.93 | 3.78 | 0.32 |  |  |  |
|  | Abnormal | 68 | -8.48 | 4.33 | 0.53 | 5.707 | -0.930 | 0.353 |
| PhNR_Tmin_0.8 (*u*V) | Normal | 140 | -9.24 | 5.43 | 0.46 |  |  |  |
|  | Abnormal | 70 | -9.95 | 3.97 | 0.47 | 5.490 | -0.982 | 0.327 |
| PhNR_Tmin_0.95 (*u*V) | Normal | 140 | -9.57 | 5.53 | 0.46 |  |  |  |
|  | Abnormal | 68 | -9.46 | 5.53 | 0.67 | 8.581 | 0.135 | 0.893 |
| PhNR_Tmin_1.114 | Normal | 140 | -9.47 | 7.76 | 0.66 |  |  |  |
| (*u*V) | Abnormal | 68 | -9.41 | 4.23 | 0.51 | 8.644 | 0.057 | 0.955 |
| PhNR_Tmin_1.204 | Normal | 140 | -10.83 | 6.29 | 0.53 |  |  |  |
| (*u*V) | Abnormal | 67 | -9.76 | 4.48 | 0.55 | 4.039 | 1.255 | 0.211 |

# **Supplementary Figure S3.** Recruitment Flowchart.

A flowchart displays the numbers of recruited cases taking part in the motion coherence and electroretinogram tests in each age groups. ASD=Autism spectrum disorder; ERG=Electroretinogram test; n=number of cases.


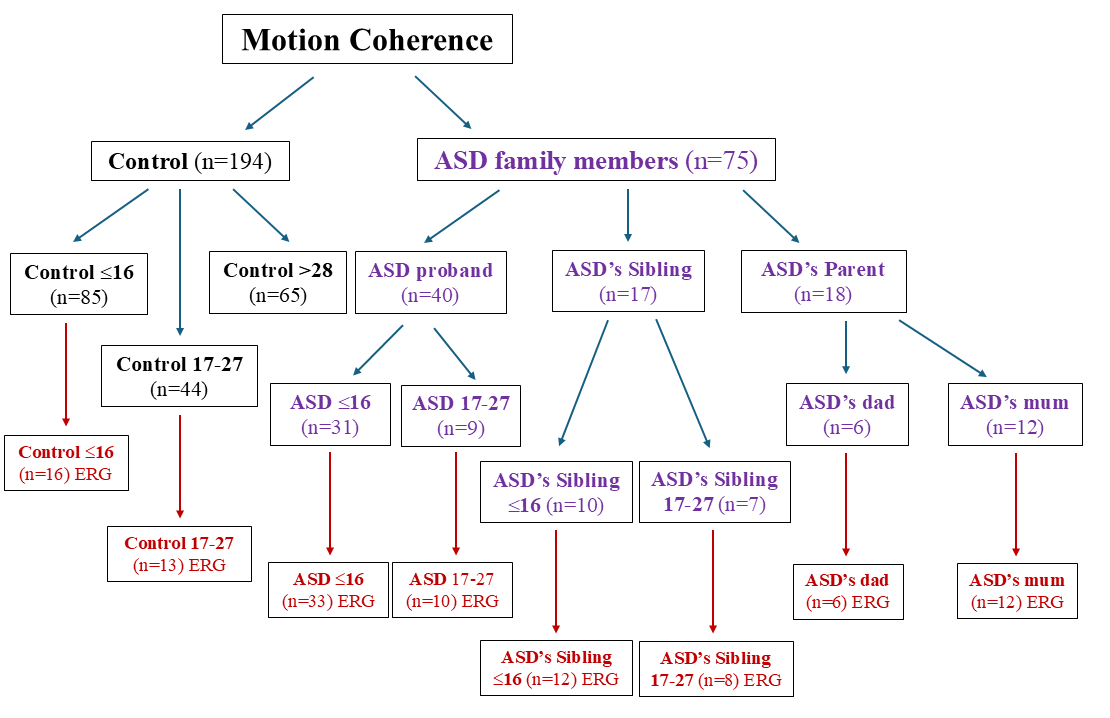


# **Supplementary Note S1.** ERG parameters

**a-, b-wave amplitudes and time (ms)-to-peak**

The standard features of a-wave were the negative trough, the amplitude from the baseline to the a-wave trough and the time (ms) from the light onset to the trough of a-wave. We presented the amplitude of a-wave in positive values to show the magnitude in the results. The characteristic of b-wave focuses on the peak-to-peak amplitude from the peak of the b-wave to the trough of the a-wave and on the time (ms) measured from the light onset to the time (ms) when the b-wave peaks.

**Photopic Negative Response (PhNR) parameters**

PhNR was measured using two methods:

p72, at t=72ms, post stimulus onset the amplitude from baseline to the waveform; and PhNR_Tmin, the ‘minimum’ PhNR amplitude was measured at the most negative point from the pre-stimulus 25ms baseline average in a time (ms) window of 55 and 95ms using the inbuilt RETeval algorithm.

Tmin, the time (ms) (Tmin) at which the ‘minimum’ PhNR amplitude occurred within the window were recommended by Frishman et al (2018) (1).

# **References**

1. Frishman L, Sustar M, Kremers J, McAnany JJ, Sarossy M, Tzekov R, Viswanathan S. ISCEV extended protocol for the photopic negative response (PhNR) of the full-field electroretinogram. Doc Ophthalmol. 2018;136(3):207-11.
